# Correlating Composition and Cation Inversion in Ternary Iron Spinel Oxides with Localization of Photoexcited States

**DOI:** 10.1021/jacs.6c05073

**Published:** 2026-06-25

**Authors:** Erica P. Craddock, William W. Brennessel, Michael T. Ruggiero, Kathryn E. Knowles

**Affiliations:** Department of Chemistry, 6927 University of Rochester, Rochester, New York 14627, United States

## Abstract

Ternary iron spinel oxides are promising materials for photo­(electro)­catalytic applications. However, like other first-row transition metal oxides, these materials have a propensity to form localized photoexcited states known as polarons that may impact their performance. In this work, we explicitly link computed electronic and vibrational structures to experimental optical dielectric and resonance Raman spectra to establish the optical polaronic properties of three different ternary iron spinel oxides: CoFe_2_O_4_, NiFe_2_O_4_ and FeNi_2_O_4_. Ternary iron spinel oxides are known to crystallize with a range of different distributions of cations among tetrahedral and octahedral sites. Here, we correlate heterogeneous broadening observed in resonance Raman spectra and sub-band gap charge transfer transitions observed in optical dielectric spectra to cation inversion. We use DFT *+ U + J* computations to demonstrate the sensitivity of charge transfer transitions to changes in cation inversion in CoFe_2_O_4_ and NiFe_2_O_4_. Experimentally, charge transfer transitions at the band-edge that arise from cation inversion exhibit strong phonon coupling in all three ternary iron spinel oxides, which is indicative of the formation of localized photoexcited states. We establish that, by changing the composition of the ternary iron spinel, the phonon mode that exhibits the strongest coupling to the band-edge absorption also changes, suggesting that the structure of photogenerated polarons in ternary iron spinel oxides can be controlled by tuning their composition. With a fundamental understanding of how composition influences the localization of photogenerated charge carriers, there are opportunities for rational material engineering to harness these carriers in photocatalytic applications.

## Graphical abstract

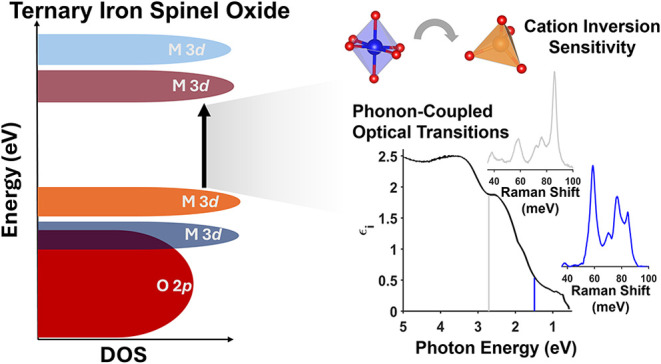

## Introduction

Ternary transition metal spinel oxides, of general formula AB_2_O_4_, demonstrate promise for photo­(electro)­chemical applications because they can accommodate a wide variety of different transition metals, their band gaps can be tuned with composition, the raw materials are often inexpensive and earth abundant, and they contain redox-active metals.^1–5^ Spinel oxides contain two different site symmetries for cations: tetrahedral (*T*_d_) and octahedral (*O*_h_). The distribution of cations among these two types of sites is described by an inversion parameter *x* (0.0 < *x* < 1.0) that quantifies the fraction of tetrahedral and octahedral sites occupied by B and A cations, respectively. The full spinel formula (A_1–*x*_B_*x*_)­[A_*x*_B_2–*x*_]­O_4_ denotes site occupation, where the parentheses indicate tetrahedral occupation and the square brackets indicate octahedral occupation. When *x* = 0.0, all the A cations are in tetrahedral sites and the spinel is considered “normal,” whereas when *x* = 1.0, all the A cations are in octahedral sites and the spinel is fully inverted. Figure 1A shows the unit cell of a prototypical ternary iron spinel oxide, CoFe_2_O_4_, with an inversion parameter of *x* = 0.0 and illustrates the characteristic Neels-type ferrimagnetic ordering in which pairwise exchange coupling results in parallel spin alignment for *O*_h_-*O*_h_ and *T*_d_-*T*_d_ pairs and antiparallel alignment for *O*_h_-*T*_d_ pairs.^6–8^ Intermediate values of *x* correspond to population of A cations in both tetrahedral and octahedral sites as shown in Figure 1B.

**Figure 1:**
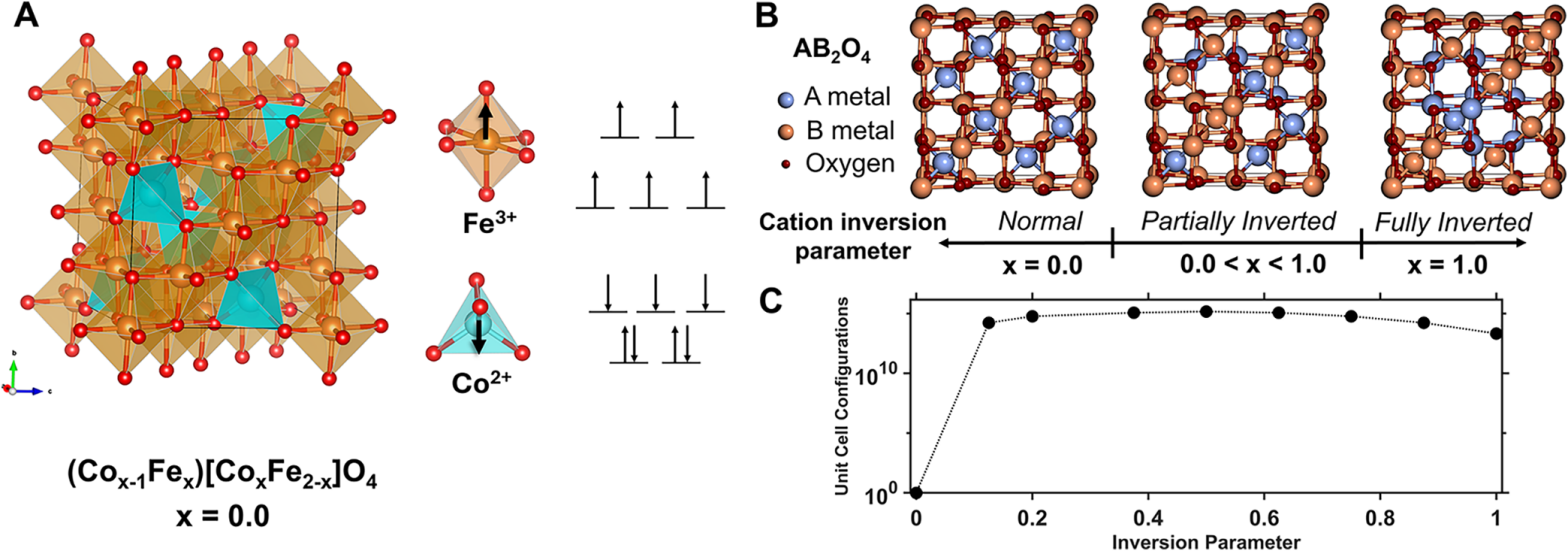
(A) Unit cell of normal CoFe_2_O_4_ (*x* = 0.0) where the orange octahedra denote Fe^3+^ ions and the blue tetrahedra denote Co^2+^ ions. The crystal field splitting diagrams of the two metal ions are shown to the right and account for the ferrimagnetic ordering: the unpaired spin on the two ion types are aligned in an antiparallel configuration. (B) Ternary spinel oxide unit cells (56 total atoms each: 8 metal A, 16 metal B, and 32 oxygen) with varying degrees of cation inversion. The partially inverted unit cell depicted has an inversion parameter of *x* = 0.375. It should be noted that both the partially and fully inverted unit cells shown represent one of many cation configurations in the unit cell that yields the denoted inversion parameter. (C) Plot showing the number of configurations possible for a ternary spinel oxide unit cell of 56 atoms for a given value of the inversion parameter; note the number of configurations is plotted on a logarithmic scale.

Ternary spinel oxides are well-known to crystallize with various degrees of cation inversion (i.e., various values of *x*),^8–14^ driven in part by the large configurational entropy present when *x* > 0. Figure 1B shows unit cells of a ternary spinel oxide with different degrees of inversion. Mathematically, there is only one unique configuration of atoms that will yield an inversion parameter of *x* = 0.0, where all *T*_d_ sites are occupied by A metals and all *O*_h_ sites are occupied by B metals. As shown in Figure 1C, there are orders of magnitude more possible arrangements when *x* > 0. Furthermore, since each inversion parameter has multiple possible configurations, in a spinel with *x* > 0 adjacent unit cells may have different configurations despite having the same overall inversion parameter. The site-by-site arrangement of cations within a ternary spinel oxide has been shown to significantly impact carrier transport pathways. Bhargava, *et al*. showed that carrier transport in ternary spinel oxides containing Mn combined with Fe or Co occurs via small polaron hopping between adjacent octahedral sites occupied by the same metal (i.e., Mn^3+^/Mn^2+^ hopping or Fe^3+^/Fe^2+^ hopping).^15,16^ The specific arrangement of cations (broadly parametrized by the cation inversion parameter *x*) determines the likelihood that adjacent octahedral sites are occupied by the same metal and therefore determines the polaron percolation pathways available in these materials.^15,16^ Here, we aim to investigate how composition and cation distribution impact optical transitions that lead to polaron formation in photoexcited ternary iron spinel oxides.

In general, there are three types of optical transitions available to metal oxides containing open-shell first-row transition metals: ligand-to-metal charge transfer (LMCT) transitions from O 2*p* to metal 3*d* bands, metal-to-metal charge transfer (MMCT) transitions between metal 3*d* bands, and intra-atomic transitions between 3*d* orbitals on the same metal ion (also referred to as ligand field transitions) (Figure 2). The energies and availabilities of these transitions depend on the relative contributions of metal 3*d* orbitals to the conduction and valence band-edge states, which is determined by the coordination geometry and 3*d* electron configuration of the metal. For example, the absorption spectrum of hematite (α-Fe_2_O_3_) is dominated by LMCT transitions because (i) the lack of Fe 3*d* character at the valence band-edge precludes MMCT transitions, and (ii) ligand field transitions are spin-forbidden in high-spin *O*_h_ Fe^3+^.^17^ In contrast, Co_3_O_4_ contains all three types of transitions, with LMCT and MMCT transitions overlapping in energy throughout the visible region and ligand field transitions arising from the *T*_d_ Co^2+^ sites appearing in the near-infrared.^18^ Previous computational reports have investigated the influence of cation inversion on the electronic structure of ternary spinel oxides.^14,19,6,20–24^ For example, the electronic structure and optical transitions of ZnFe_2_O_4_ were assessed as a function of inversion by Fritsch.^19^ Although the band gap of ZnFe_2_O_4_ was found to remain largely unchanged, multiple LMCT transitions become allowed when both *O*_h_ and *T*_d_ sites are occupied by Fe.^19^ Likewise, in the ternary spinel oxide NiCo_2_O_4_, Cheng, *et al*. show that the distribution of 3*d* metal character across the band-edge changes with inversion.^6^ With both Ni and Co 3*d* orbitals contributing to the band-edge electronic character, not only do the energies of optically allowed transitions vary with cation inversion, but also the electronic character of the bands participating in localized MMCT transitions demonstrates sensitivity to cation inversion.

**Figure 2:**
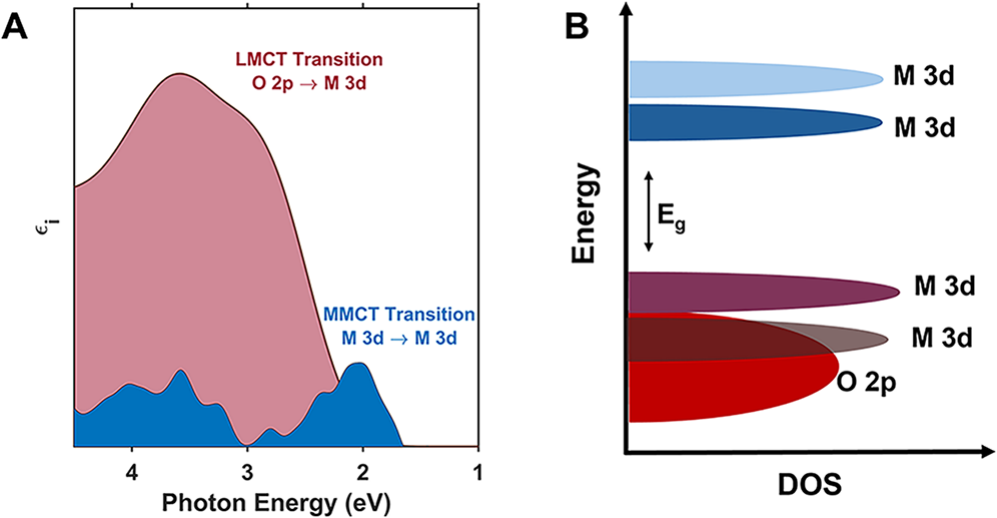
(A) Schematic depiction of the ligand to metal charge transfer (O 2*p* → M 3*d*) and metal to metal charge transfer (M 3*d* → M 3*d*) contributions to the imaginary dielectric spectrum of ternary spinel oxide materials and (B) the associated electronic density of states diagram.

Selective excitation of these different types of transitions (e.g., LMCT vs. MMCT) can produce different types of excited states, including polaronic excited states, that exhibit different dynamics and decay pathways. Our group has shown that excitation of the lowest energy LMCT transition in α-Fe_2_O_3_ and the lowest energy MMCT transition in Co_3_O_4_ results in direct population of polaronic excited states.^18,25,26^ Other groups have shown that higher energy excitations of LMCT transitions in α-Fe_2_O_3_, Co_3_O_4_, or NiFe_2_O_4_ and a higher energy MMCT state in CoFe_2_O_4_ lead to rapid relaxation into excited states in which one or both carriers have formed small polarons.^27–31^ In ternary iron spinel oxides, this polaronic excited state resembles an MMCT state with the electron localized on an octahedral Fe site and the hole localized on an octahedral Ni or Co site.^27,28^ Restelli *et al.* showed that excitation of the higher energy LMCT transition in Co_3_O_4_ results in coherent scattering of a different phonon than is observed upon excitation of the lower energy MMCT transition, indicating that different phonons couple to these optical transitions.^29^ Additionally, ultrafast optical nanoscopy combined with terahertz spectroscopy studies of Co_3_O_4_ demonstrates that photoexcitation of an MMCT transition yields a higher photocarrier diffusion constant that that obtained upon photoexcitation of an LMCT transition.^32^ Furthermore, the presence of optically accessible ligand field states has been posited to provide a pathway for rapid nonradiative decay to the ground state.^33^ Formation of polaron states and rapid relaxation through ligand field manifolds have both been implicated as inhibiting the efficient extraction of charge carriers from photoexcited transition metal oxides, a process that is crucial to their performance as photoelectrocatalysts.^33,34^

Given the previously established connections between cation distribution and the nature of optical transitions in ternary spinel oxides (e.g., LMCT vs. MMCT), here we explicitly link computed electronic structures of ternary iron spinel oxides CoFe_2_O_4_ and NiFe_2_O_4_ with various degrees of cation inversion to experimental optical dielectric spectra collected for polycrystalline thin films of these materials. This comparison enables us to assign the observed optical transitions to LMCT or MMCT processes. Comparing the experimental dielectric spectrum of NiFe_2_O_4_ to that of FeNi_2_O_4_ enables us to compare stoichiometry and its influence on the relative intensities of optical transitions. We further examine the role of LMCT vs. MMCT transitions in mediating photoinduced polaron formation using a combined approach of resonance Raman spectroscopy and DFT*+U+J*-computed phonon modes. We investigate the influence of band-edge orbital composition and cation inversion on optoelectronic properties by comparing the Stokes resonance Raman spectra of CoFe_2_O_4_, NiFe_2_O_4_ and FeNi_2_O_4_, all of which have Raman spectra exhibiting heterogeneous broadening due to cation inversion. Using Stokes resonance Raman spectroscopy, we assess changes in the resonance enhancement of phonon modes of ternary iron spinel oxides as a function of excitation photon energy. We find evidence of localized electronic excited states that are strongly coupled to specific phonons and accessed via excitation of transitions at the optical band-edge that arise from cation inversion. We show that changing the composition of the ternary iron spinel oxide changes which phonon mode exhibits the strongest coupling to the band-edge absorption, suggesting that the structure of photogenerated polarons in iron spinel oxides can be controlled by tuning their composition.

## Results and Discussion

### Structural Characterization of Ternary Iron Spinel Oxides

We fabricated polycrystalline thin films of CoFe_2_O_4_, NiFe_2_O_4_, and FeNi_2_O_4_ of exceptionally high optical quality using a sol–gel process (see Supporting Information for Experimental Methods). Powder X-ray diffraction patterns of these films reveal highly crystalline, phase-pure spinel materials (Figure 3B). The lattice parameters were determined with Rietveld refinement (Table 1 and Figure S7). The metal/metal ratios calculated from the relative intensities of K_α_ emission peaks observed for the respective metals in X-ray fluorescence spectra of the films demonstrate near-perfectly stoichiometric B/A ratios of 2:1 for all three materials (Table 1, spectra shown in Supporting Information, Figure S6). Methods for quantifying cation inversion in ternary iron spinel oxides include tracing the oxidation state and coordination environment of the individual metals using X-ray photoelectron spectroscopy (XPS),^35^ high-resolution X-ray emission spectroscopy,^36^ or ^57^Fe Mössbauer spectroscopy.^8^ Other characterization techniques, such as magnetization curves^37^ and Rietveld refinement of neutron diffraction patterns,^13^ can also offer insights into cation distribution of ternary spinel oxides. Here, we are focused on assessing the optical properties of polycrystalline spinel oxide thin films, which requires the use of nonconductive substrates of high optical quality (e.g., fused quartz and sapphire). XPS requires the use of a conductive substrate (typically silicon wafers), Mössbauer and magnetism studies require substantial amounts of powdered samples, and high-resolution X-ray emission and neutron diffraction measurements require access to large-scale user facilities. Thus, we use Raman spectroscopy, a technique accessible to a standard academic laboratory, to assess cation inversion in our samples.

**Figure 3:**
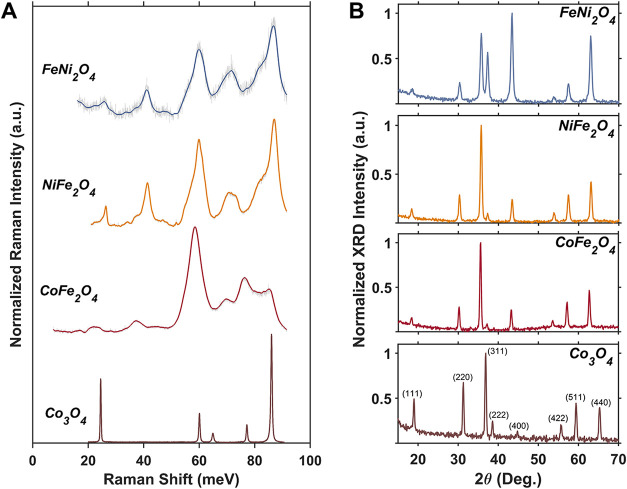
Low-frequency Stokes resonance Raman spectra (A) and powder XRD diffraction patterns (B) of polycrystalline thin films of transition metal spinel oxides annealed on sapphire substrates. From top to bottom: FeNi_2_O_4_, NiFe_2_O_4_, CoFe_2_O_4_ and Co_3_O_4_. The Raman spectra were collected with a laser excitation of 1.58 eV. The Co_3_O_4_ pXRD pattern has labeled Miller indices according to ICCD:04–016–4508. The Raman spectrum of Co_3_O_4_ is reproduced from ref 18. Available under a CC BY 3.0 license. Copyright 2025 Royal Society of Chemistry.

**Table 1:** Structural Parameters of Polycrystalline Spinel Oxide Thin Films Fabricated on Sapphire Substrates

Spinel Oxide	Lattice Parameter (Å)	Metal B K_α_:Metal A K_α_
CoFe_2_O_4_	8.376	1.948 ± 0.003
NiFe_2_O_4_	8.336	1.895 ± 0.001
FeNi_2_O_4_	8.341	2.006 ± 0.003
Co_3_O_4_	8.058	-

Raman spectroscopy reveals the energies of the phonon modes, which are sensitive to metal occupation, and elucidates the translational symmetry of the crystal. Importantly, changes in configuration from unit cell to neighboring unit cell that occur in inverted or partially inverted ternary spinel oxides break the translational symmetry and manifest in the Raman spectrum as heterogeneous broadening (Figure 3A). Figure 3A overlays Stokes resonance Raman spectra of polycrystalline thin films of the different iron spinel oxide materials with a Stokes resonance Raman spectrum of the binary spinel oxide Co_3_O_4_. The iron spinel materials have Raman features centered at similar frequencies to Co_3_O_4_; however, the peaks are significantly broader than those in Co_3_O_4_. The Raman spectrum of Co_3_O_4_ has five distinct Raman peaks, which follows the foundational calculations by White and DeAngelis demonstrating that a normal spinel has five Raman active modes.^38^ With a decrease in symmetry resulting from cation inversion, Raman selection rules dictate an increase in the number of Raman active modes.^38^ All the ternary iron spinel oxides have a Raman peak at ∼40 meV that is not present in Co_3_O_4_, indicating they all crystallized with some degree of cation inversion. The phenomenon of increased number of Raman modes with inversion has been observed in other ternary spinel oxides.^18,39^ Despite the broadened Raman spectra observed for the ternary iron spinel oxides, the corresponding pXRD patterns of the spinel oxides shown in Figure 3B all indicate a high degree of crystallinity, with all samples indexing to the *Fd*3̅*m* space group. The similar ionic radii of the cations in this study (Fe^3+^= 91 pm, Co^2+^ = 89 pm, Ni^2+^ = 84 pm)^40^ enable cation inversion while maintaining high crystallinity and similar lattice parameters (Table 1).

The broadening in the Raman features observed in the iron spinel oxides therefore cannot be attributed to poor sample crystallinity or phase impurities, but rather to a decrease in translational symmetry that leads to heterogeneous broadening in the energies of the phonon modes due to variations in the reduced mass of the modes arising from local disorder. For example, upon comparing the Raman spectra of the ternary iron spinel oxides to Co_3_O_4_, it is evident that the Raman features peaking at ∼86 meV in both NiFe_2_O_4_ and FeNi_2_O_4_ have a lower energy shoulder, which is not present in the 86-meV mode of Co_3_O_4_. The 86-meV phonon mode in Co_3_O_4_ corresponds to a symmetric “breathing mode” of the oxygen atoms around tetrahedral sites.^18^ The lower energy shoulder in both Fe–Ni spinel oxides arises primarily from the occupation of the tetrahedral sites by two different metals (Fe vs. Ni) resulting in two distinguishable peaks. The ∼60-meV phonon mode, which corresponds to motion of the metals occupying octahedral sites in Co_3_O_4_, also exhibits lower energy shoulders in NiFe_2_O_4_ and FeNi_2_O_4_. These shoulders are not as distinct in CoFe_2_O_4_, likely due to the greater similarity of atomic mass between Co and Fe compared to Ni and Fe.

### Optical Transitions and Electronic Structure of Ternary Iron Spinel Oxides

The computed imaginary dielectric spectrum of normal CoFe_2_O_4_ (*x* = 0.0) is shown in Figure 4A, overlaid with the experimentally determined spectrum of CoFe_2_O_4_; the associated spin-resolved projected densities of states of normal CoFe_2_O_4_ (*x* = 0.0) are shown in Figure 4B. The band edges of normal CoFe_2_O_4_ are dominated by *T*_d_ Co character in both the valence and conduction bands in the spin-up channel while the spin-down channel is dominated by *O*_h_ Fe in the conduction band and a combination of *T*_d_ Co and oxygen in the valence band. With this orbital composition, LMCT transitions from O 2p → *O*_h_ Fe (spin down), and MMCT transitions from *T*_d_ Co → *O*_h_ Fe (spin down) and *T*_d_ Co → *T*_d_ Co (spin up) constitute the optical spectrum of normal CoFe_2_O_4_. However, this computed spectrum fails to capture the low energy (*E* < 1.5 eV) features present in the measured spectrum. We therefore assessed other computed degrees of inversion to best match the experimental dielectric spectrum (Figure 4). Fully inverted CoFe_2_O_4_ (*x* = 1.0) has a narrower band gap than that of normal CoFe_2_O_4_ (*x* = 0.0), with an absorption onset at 0.9 eV compared to 1.8 eV, respectively (Figure 4A,E). The smaller band gap of fully inverted CoFe_2_O_4_ can be explained by the change in orbital composition at the band-edge: the spin-down channel is dominated by *O*_h_ Co character in the valence band and *O*_h_ Fe character in the conduction band, with a corresponding energy gap matching 0.9 eV. With changes to the occupation of the cations in CoFe_2_O_4_, a new MMCT transition becomes optically available (*O*_h_ Co → *O*_h_ Fe, spin down) and the band gap decreases. Additionally, the spin-up channel of fully inverted CoFe_2_O_4_ is dominated by an LMCT from O 2p → *T*_d_ Fe and an MMCT from *O*_h_ Co → *T*_d_ Fe, further indicating that new charge transfer transitions emerge with changes to cation inversion.

**Figure 4:**
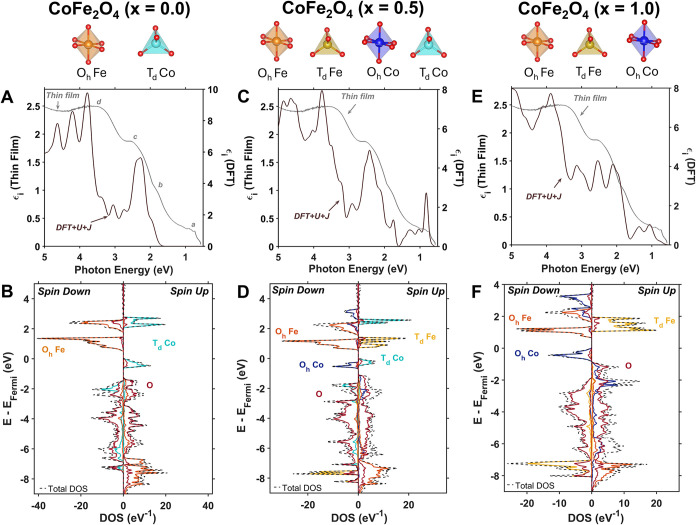
DFT + *U + J-*computed imaginary dielectric spectrum of (A) normal CoFe_2_O_4_ (*x* = 0.0), (C) partially inverted CoFe_2_O_4_ (*x* = 0.5) and (E) fully inverted CoFe_2_O_4_ (*x* = 1.0). The metal geometries in each inversion configuration are shown above the respective dielectric spectrum. All computed imaginary dielectric spectra (maroon) are overlaid with the experimental imaginary dielectric spectrum (gray). The associated projected densities of state are shown for (B) normal CoFe_2_O_4_ (*x* = 0.0), (D) partially inverted CoFe_2_O_4_ (*x* = 0.5) and (F) fully inverted CoFe_2_O_4_ (*x* = 1.0).

The electronic structure of CoFe_2_O_4_ with partial inversion (*x* = 0.5) has attributes of both normal and fully inverted CoFe_2_O_4_, indicative of the complexities that emerge in electronic structure with cation inversion (Figure 4D). The transitions in the spin-up channel map onto *T*_d_ Co → *T*_d_ Fe and O 2p → *T*_d_ Fe, and the transitions in the spin-down channel map onto *O*_h_ Co → *O*_h_ Fe and O 2p → *O*_h_ Fe. We note that, within the spinel lattice, adjacent tetrahedral cation sites are separated by an octahedral site (Figure 1). Thus, an MMCT from *T*_d_ Co to *T*_d_ Fe is likely to have very low transition probability due to small electronic overlap.^15,16^ The computed dielectric spectrum shown in Figure 4C shows that computed low-energy transitions in partially inverted CoFe_2_O_4_ (*x* = 0.5) match the transitions observed experimentally. We note that literature reports indicate that the thermodynamically favored inversion parameter of CoFe_2_O_4_ is *x* = 0.7;^8^ however, this exact degree of inversion could not be implemented in electronic computations without imposing inaccurate strain in the supercell structure. Nevertheless, the match between experimental and computational dielectric spectra indicates the experimental CoFe_2_O_4_ sample contains substantial populations of *T*_d_ and *O*_h_ sites occupied by both Co and Fe. With both spin channels dominated by metal 3*d* character in CoFe_2_O_4_ (*x* = 0.5), more MMCT transitions are allowed in addition to LMCT transitions. Table 2 summarizes the assignments of various transitions in the dielectric spectrum of partially inverted CoFe_2_O_4_. Importantly, the features with energies of 2.4 eV and below (a–c) are assigned to a combination of overlapping MMCT and LMCT transitions whereas the more intense and higher energy feature at 3.4 eV (d) is assigned to LMCT transitions. There are both interatomic MMCT and intra-atomic ligand-field type transitions contributing to oscillator strength. The intra-atomic ligand-field transitions are more localized as they occur within the same atom whereas interatomic transitions involve charge transfer between two different metal sites. Note that transitions within both the spin-up and spin-down channels contribute to both the MMCT and LMCT regions of the spectrum. (see Supporting Information for details about DFT+*U+J* methods.)

**Table 2:** Assignment of Optical Transitions in CoFe_2_O_4_

Peak Center (eV)	Peak Label	Transition Assignment	Transition Type
0.8	a	*O*_h_ Co → *O*_h_ Fe (spin down)	MMCT
		*T*_d_ Co → *T*_d_ Fe (spin up)	MMCT
1.5	b	*O*_h_ Co → *O*_h_ Fe (spin down)	MMCT
		O 2p → *T*_d_ Fe (spin up)	LMCT
2.4	c	*T*_d_ Co → *O*_h_ Fe (spin down)	MMCT
		O 2p → *O*_h_ Fe (spin down)	LMCT
		*T*_d_ Co → *T*_d_ Co (spin up)	intra-atomic ligand field
3.4	d	O 2p → *O*_h_ Fe (spin down)	LMCT
		O 2p → *T*_d_ Co (spin up)	LMCT

Upon replacing Co with Ni, we observe that the experimental imaginary dielectric spectrum of NiFe_2_O_4_ (Figure 5A) has a steeper onset of band-edge absorption at a higher energy (1.8 eV) compared to CoFe_2_O_4_ (absorption onset at ∼0.5 eV). However, there are still weak, narrow features apparent in the dielectric spectrum of NiFe_2_O_4_ both below and within the broad continuum absorption. These features occur at 1.6 eV (a), 1.9 eV (b), 2.1 eV (c) and 2.5 eV (d) (Figure 5A). Although previous literature shows NiFe_2_O_4_ is prone to crystallize with an inversion parameter of *x* = 1.0 under thermodynamic control, the computed dielectric spectrum of fully inverted NiFe_2_O_4_ did not predict the 1.6-eV transition observed experimentally (see Figure S2 in the Supporting Information). The imaginary dielectric spectrum of NiFe_2_O_4_ with an inversion parameter of *x* = 0.5 was calculated using DFT+*U*+*J* and has good agreement with the experimentally determined spectrum (Figure 5A). The projected densities of states of NiFe_2_O_4_ (*x* = 0.5) are similar to those of CoFe_2_O_4_ (*x* = 0.5), as the distribution of Fe 3*d* character is almost identical between the two materials, indicating the primary LMCT-type transition from O 2*p* to Fe 3*d* is largely unchanged (Figure 5B). The main differences between NiFe_2_O_4_ and CoFe_2_O_4_ are the energetic positions of the A metal electronic character at the band edge, which give rise to the unique MMCT transitions that occur in each ternary iron spinel. More specifically, in NiFe_2_O_4_ the MMCT transitions occur at higher energies than those observed in CoFe_2_O_4_ (Tables 2 and 3).

**Figure 5:**
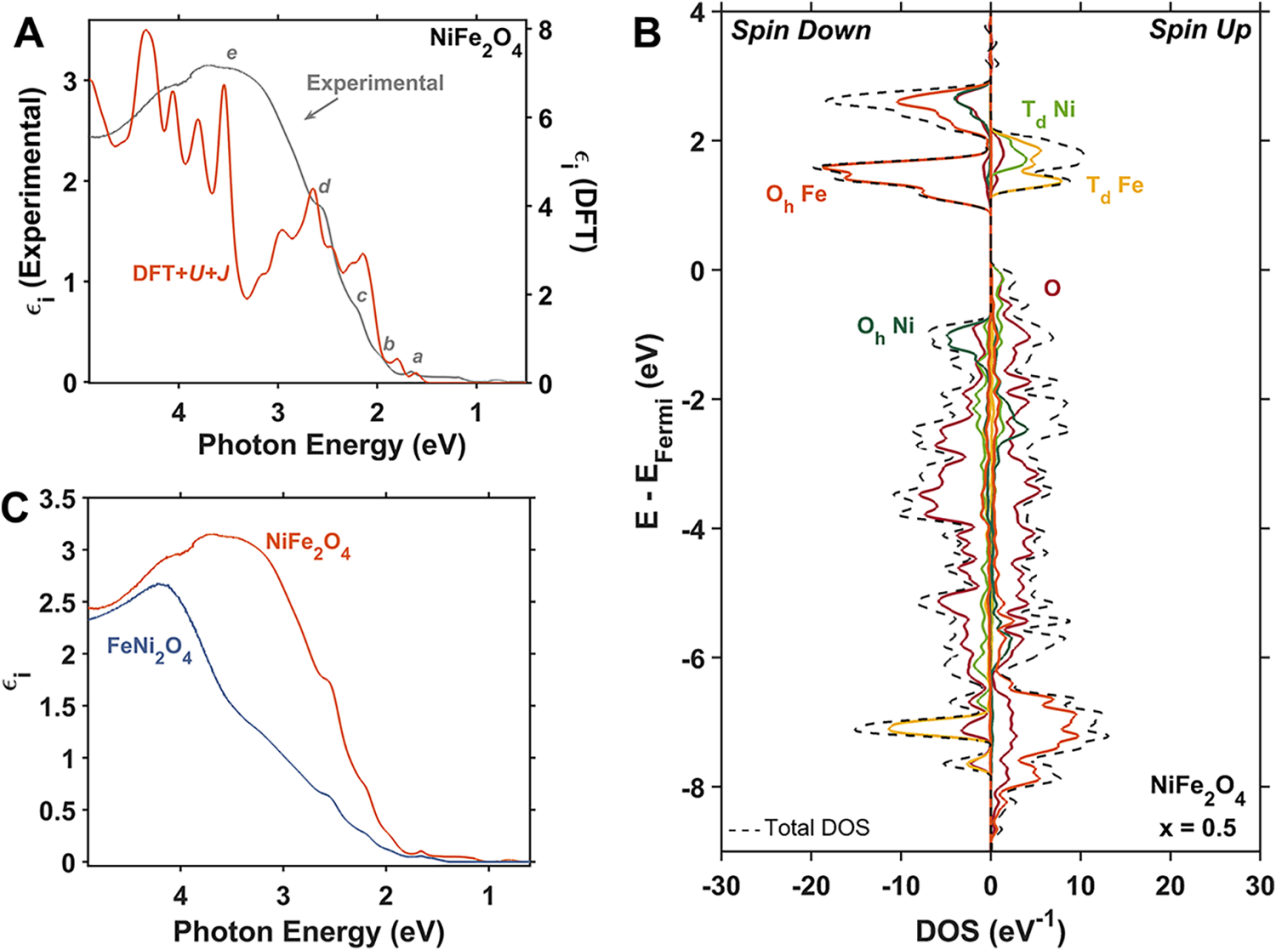
(A) Imaginary dielectric spectrum of NiFe_2_O_4_ experimentally determined (gray) and computed from DFT+*U*+*J* with a partially inverted primitive cell (*x* = 0.5) (orange). (B) Projected electronic densities of states of partially inverted NiFe_2_O_4_ (*x* = 0.5). (C) Imaginary dielectric spectrum of NiFe_2_O_4_ (orange) overlaid with that of FeNi_2_O_4_ (blue); both spectra are experimentally determined.

**Table 3:** Assignment of Optical Transitions in NiFe_2_O_4_

Peak Center (eV)	Peak Label	Transition Assignment	Transition Type
1.6	a	*T*_d_ Ni → *T*_d_ Fe (spin up)	MMCT
		O 2p → *T*_d_ Fe (spin up)	LMCT
1.9	b	*T*_d_ Ni → *T*_d_ Ni (spin up)	intra-atomic ligand field
		O 2p → *T*_d_ Ni (spin up)	LMCT
2.1	c	*O*_h_ Ni → *O*_h_ Fe (spin down)	MMCT
		O 2p → *O*_h_ Fe (spin down)	LMCT
2.5	d	*O*_h_ Ni → *O*_h_ Fe (spin down)	MMCT
		O 2p → *O*_h_ Fe (spin down)	LMCT
3.5	e	O 2p → *O*_h_ Fe (spin down)	LMCT

Exchanging Co for Ni in the iron spinel oxide (AFe_2_O_4_) structure contributed to changes in the energies of localized MMCT transitions at the band-edge, leading to changes in the energies at which narrow optical features are observed. Conversely, upon changing the stoichiometry from NiFe_2_O_4_ to FeNi_2_O_4_, we primarily observe changes in the relative intensities of optical transitions (Figure 5C). The dielectric spectrum of FeNi_2_O_4_ has weak features at the same energies as features observed in NiFe_2_O_4_, implying the change in Ni/Fe stoichiometry does not influence the energies of MMCT transitions. Instead, we primarily observe a decrease in the intensity of the LMCT transitions. The MMCT transitions in NiFe_2_O_4_ are associated with both metals in each coordination geometry (*T*_d_ Fe, *O*_h_ Fe, *T*_d_ Ni and *O*_h_ Ni, Table 3), suggesting that FeNi_2_O_4_ must also be partially inverted to exhibit the same MMCT transitions as NiFe_2_O_4_. Additionally, the Raman features of NiFe_2_O_4_ and FeNi_2_O_4_ shown in Figure 2A are nearly identical, further indicating that they have similar degrees of cation inversion.

### Optical Polaronic Properties of Ternary Iron Spinel Oxides

CoFe_2_O_4_ (*x* = 0.5) and NiFe_2_O_4_ (*x* = 0.5) have characteristics of both α-Fe_2_O_3_ and Co_3_O_4_: Fe 3*d* character dominating the conduction band-edge and localized MMCT transitions, respectively (see Figures S1 and S3 in Supporting Information for full band structures). These fundamental properties of α-Fe_2_O_3_ and Co_3_O_4_ are associated with photoinduced polaron formation.^18,25,26,41^ Previous optical pump-XUV probe measurements of partially inverted CoFe_2_O_4_ and NiFe_2_O_4_ reveal relaxation into excited states containing electron polarons localized at Fe centers and hole polarons localized around Co or Ni centers, respectively; these states resemble MMCT excited states.^27,28^ Here, we employ Stokes resonance Raman spectroscopy to investigate the coupling between various optical transitions in ternary iron spinel oxides and specific phonon modes. We note that although our resonance Raman approach provides information about which specific phonons couple to which specific optical transitions to enable photoinduced polaron formation, it does not provide precise insights into which charge is being localized. Furthermore, polaronic distortions typically involve contributions from several different phonon modes; however, previous work from our group on hematite demonstrates that tracing resonance Raman intensity with excitation wavelength provides insights into which phonon modes contribute the largest displacement to polaronic distortions.^25,26^

We collected Stokes resonance Raman spectra using excitation photon energies that span the dielectric spectrum of each ternary iron spinel oxide. Figure 6A plots a selection of resonance Raman spectra of CoFe_2_O_4_ collected at various excitation photon energies and internally normalized to the most intense feature. The remaining resonance Raman spectra are shown in the Supporting Information (Figure S13). We observe that the relative intensities of the 86- and 58-meV phonon modes shift depending on the excitation energy: the 86-meV phonon mode is most intense when excited at 2.71 eV and the 58-meV phonon mode is most intense when excited at 1.49 eV (Figure 6A). This shift in phonon intensity in CoFe_2_O_4_ with excitation photon energy is evidence that these phonons couple to different optical transitions. From the projected density of states of CoFe_2_O_4_, it is clear the optical transition at 2.71 eV is associated with an LMCT-type transition from O 2*p* to Co 3*d* orbitals (Figure 4C,D). The optical transition at 1.49 eV is assigned to the MMCT of *O*_h_ Co → *O*_h_ Fe (spin down) (Table 2). Figure 6B plots the intensities of each Raman mode versus excitation photon energy; these intensities are corrected for wavelength-dependent scattering efficiency and absorption cross-section of the CoFe_2_O_4_ film. These corrected intensities are overlaid with the experimental imaginary dielectric spectrum collected for CoFe_2_O_4_. Intriguingly, when comparing the corrected Raman intensities to the sample absorption spectrum, it becomes apparent that with excitation between 1.58 and 1.49 eV, there is significant enhancement of all Raman signals, especially the one corresponding to the 58-meV phonon (Figure 6B). The magnitude of the imaginary dielectric spectrum in the region of 1.58–1.49 eV is lower than that of any other Raman excitation photon energies used, yet the Raman signal is most enhanced at this excitation energy. The phonon displacement vectors of CoFe_2_O_4_ (*x* = 0.5) were calculated using DFT+*U*+*J*. The 58-meV phonon mode is associated with the *O*_h_ sites breathing relative to one another (Figure 6C). The assignment of the optical transition at ∼1.5 eV to MMCT from *O*_h_ Co → *O*_h_ Fe, is consistent with the observation that the phonon mode enhanced at this excitation energy is associated with motion of the *O*_h_ sites.

**Figure 6:**
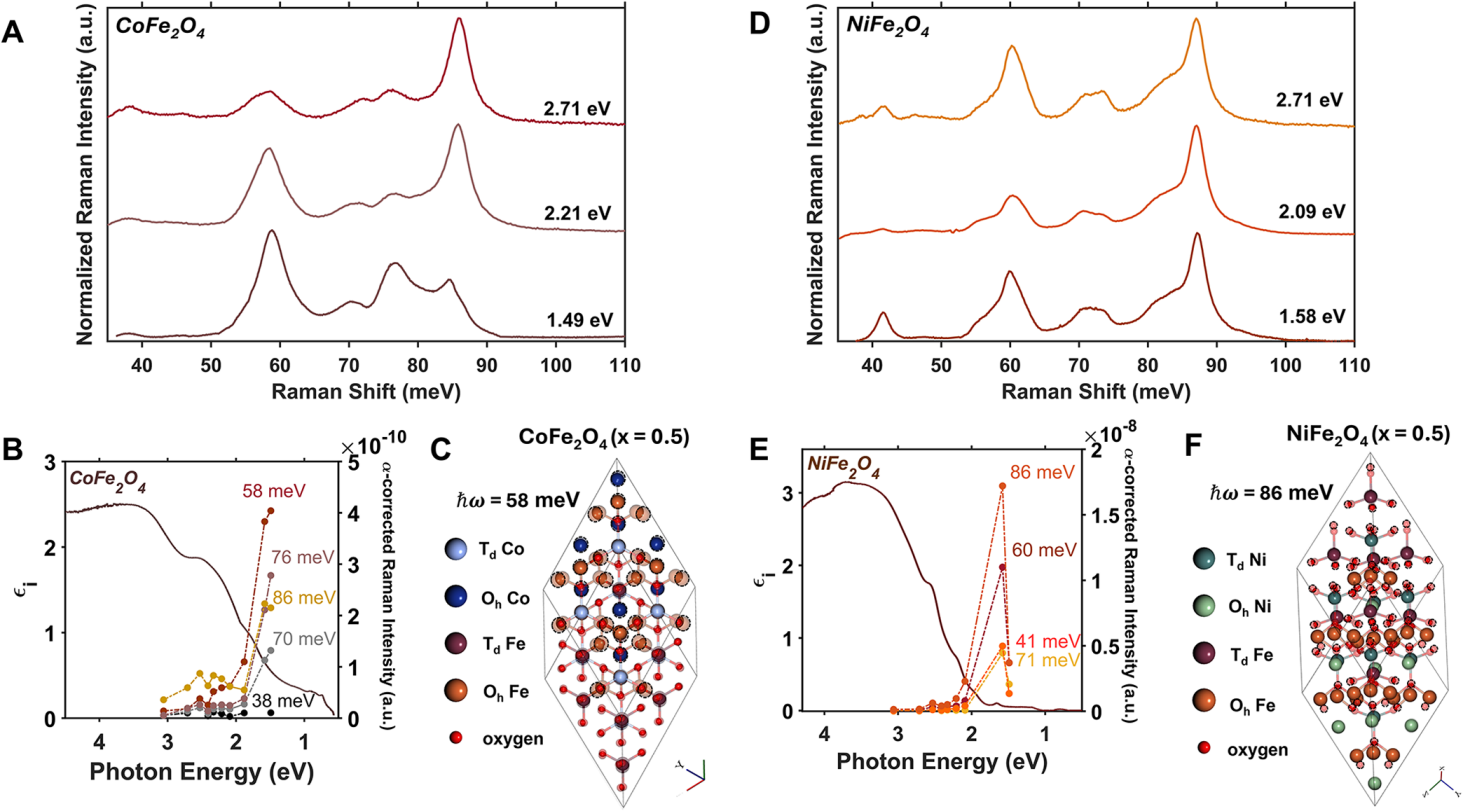
Internally normalized Stokes resonance Raman spectra of (A) CoFe_2_O_4_ and (D) NiFe_2_O_4_ polycrystalline thin films. Excitation photon energies of 2.71, 2.21, and 1.49 eV are shown for CoFe_2_O_4_ and 2.71, 2.09, and 1.58 eV for NiFe_2_O_4_. Imaginary dielectric spectrum overlaid with Raman mode intensities corrected for scattering cross section and sample absorption of (B) CoFe_2_O_4_ and (E) NiFe_2_O_4_. Phonon displacement vectors of (C) CoFe_2_O_4_ associated with the 58-meV mode and (F) NiFe_2_O_4_ associated with the 86-meV mode.

The relative changes in Raman modes with excitation energy in NiFe_2_O_4_ are more subtle than those observed in CoFe_2_O_4_. The 86-meV phonon mode in NiFe_2_O_4_ is most intense across the entire dielectric spectrum and the 60-meV phonon mode consistently has the second highest relative intensity (Figures 6D and S11B). When considering the Raman intensities corrected for scattering cross section and sample absorption, the Raman spectrum of NiFe_2_O_4_ is most enhanced when excited at 1.58 eV, and the 86-meV phonon is the most intense feature (Figure 6E). The phonon displacement vector of the 86-meV phonon was calculated using DFT+*U*+*J* on a partially inverted primitive cell of NiFe_2_O_4_ and is associated with oxygen breathing about *T*_d_ sites (Figure 6F). Based on the electronic structure and dielectric spectrum of NiFe_2_O_4_, the optical feature at 1.58 eV results from transitions into a *T*_d_ Fe band (Table 3). Thus, we again observe a correlation between the geometry of the metal sites contributing to the optical transitions at which the maximal Raman enhancement is observed and the geometry of the phonon mode that exhibits the strongest enhancement.

To assess the influence of changes to metal stoichiometry on phonon coupling to optical transitions, we performed analogous resonance Raman analysis on FeNi_2_O_4_. We observe that FeNi_2_O_4_ also demonstrates strong resonance enhancement of the 86-meV phonon when excited at 1.49 eV (Figure S10). The similar nature of the resonance Raman enhancement in FeNi_2_O_4_ compared to NiFe_2_O_4_ indicates that the localized charge transfer transitions that become allowed with cation inversion in Fe–Ni ternary spinel oxides drive phonon coupling at the band edge, and this phonon coupling does not strongly depend on the Fe–Ni stoichiometry. Resonance Raman analysis of CoFe_2_O_4_, NiFe_2_O_4_ and FeNi_2_O_4_ samples annealed on quartz yields identical trends of phonon mode enhancement as observed for the samples annealed on sapphire substrates (see Supporting Information, Figure S15), indicating that the substrate does not impact these observed trends.

Overall, our analysis of the resonance Raman excitation profiles and the geometries of the phonon modes that exhibit the strongest resonance enhancement strongly suggest that cation inversion dictates phonon coupling to optical transitions in ternary iron spinel oxides. The optical transitions that exhibit the strongest phonon coupling involve bands that are the direct result of cation inversion, namely *O*_h_ Co in CoFe_2_O_4_ and *T*_d_ Fe in NiFe_2_O_4_. Thermal difference spectra collected at elevated temperatures for CoFe_2_O_4_, NiFe_2_O_4_, and FeNi_2_O_4_ exhibit highly structured derivative lineshapes at energies coinciding with the absorption onsets (where maximal resonance enhancement occurs in the Raman spectra, see Supporting Information Figure S16). This observation indicates that the band-edge transitions arising from cation inversion are the primary contributors to the observed temperature dependence. The mechanism by which temperature impacts the intensity of optical transitions is *via* coupling to thermally populated phonons.^25^ Therefore, the significant temperature sensitivity of the band-edge transitions supports our overall conclusion that localized transitions arising from cation inversion mediate photoinduced polaron formation in ternary iron spinel oxides.

Our observation that resonance enhancement of specific phonon modes increases upon photoexcitation of band-edge transitions associated with cation inversion is consistent with our recent work comparing Co_3_O_4_ and slightly inverted ZnCo_2_O_4_.^18^ In that study, replacing an open-shell transition metal (i.e., Co) with a closed-shell metal (i.e., Zn) enabled us to gain further insights into the role of open-shell transition metals in mediating photoinduced polaron formation. In particular, the replacement of most of the tetrahedral Co atoms with Zn atoms caused the phonon mode that coupled most strongly to the band-edge MMCT transitions to shift from a tetrahedral mode to an octahedral mode. ZnFe_2_O_4_ offers the potential to conduct a similar study for ternary iron spinel oxides. The electronic structure of ZnFe_2_O_4_, a material that is somewhat analogous to ZnCo_2_O_4_, has a conduction band-edge dominated by Fe 3*d* bands and a valence band-edge dominated by O 2*p* bands both when it has a normal cation distribution and with various degrees of inversion (see Supporting Information Figures S4-S5).^19^ Optical selection rules forbid ligand field transitions within *O*_h_ Fe^3+^, and Zn^2+^ contributes minimally to the band edge due to the full occupation of its *d-*bands; therefore there are no *d–d* transitions predicted for ZnFe_2_O_4_ and the absorption spectrum is dominated by LMCT transitions.^19,42^ We therefore predict normal ZnFe_2_O_4_ (*x* = 0.0) to have similar optical polaronic characteristics to hematite due to the predominance of the LMCT transitions, with excitation at the optical band-edge leading to carrier localization centered around *O*_h_ Fe^3+^ sites. ZnFe_2_O_4_ demonstrates poor photocarrier mobility as determined by time-resolved microwave conductivity, which would be consistent with photoinduced polaron formation.^43^ Unfortunately, our attempts to synthesize thin films of ZnFe_2_O_4_ of the high optical quality required for resonance Raman measurements and determination of the dielectric spectrum consistently resulted in the formation of hematite impurities, so we were unable to include ZnFe_2_O_4_ in our experimental study. Although others have observed changes in relative intensities of optical transitions in ZnFe_2_O_4_ with inversion,^44^ CoFe_2_O_4_ and NiFe_2_O_4_, with two open-shell transition metal cations present in the lattice structure, demonstrate different optical band gaps and unique optical transitions with changes in cation distribution. The sensitivity of MMCT transitions in ternary iron spinel oxides containing two open-shell transition metals to cation inversion provides opportunities to engineer polaronic pathways.

## Conclusion

We present evidence of localized photoexcited states in ternary iron spinel oxides mediated by charge transfer transitions that arise due to cation inversion. The Raman spectra of the three ternary iron spinel oxides, CoFe_2_O_4_, NiFe_2_O_4_ and FeNi_2_O_4_, have broader features than the Raman spectrum of the binary spinel oxide Co_3_O_4_ due to the significant configurational disorder arising from cation inversion in ternary spinel oxides. Cation inversion not only disrupts the translational symmetry of the crystal, as observed in the Raman spectra, but also impacts the orbital composition at the band-edge and the nature of the optical transitions. Computations using DFT+*U+J* of CoFe_2_O_4_ and NiFe_2_O_4_ demonstrate that different degrees of inversion give rise to different types of MMCT transitions with different energies. By comparing the experimentally and computationally determined imaginary dielectric spectra, the thin film samples of CoFe_2_O_4_ and NiFe_2_O_4_ are determined to be partially inverted with band-edge absorptions dominated by transitions involving bands that are a direct result of cation inversion (i.e., *O*_h_ Co and *T*_d_ Fe, respectively). Resonance Raman spectra establish that maximal resonance enhancement occurs when the excitation photon energy matches these inversion-induced band-edge transitions. Furthermore, the displacement vectors corresponding to the phonons that exhibit the strongest resonance enhancement involve motion centered around these same *O*_h_ Co and *T*_d_ Fe centers. In CoFe_2_O_4_, a phonon mode corresponding to motion around *O*_h_ sites is most enhanced in association with an *O*_h_ Co → Fe *O*_h_ transition, whereas a *T*_d_ phonon is most enhanced upon excitation into an Fe *T*_d_ state in NiFe_2_O_4_. Upon comparing the Raman and optical spectra of FeNi_2_O_4_ and NiFe_2_O_4_, it is evident that the Ni/Fe ratio is not crucial for mediating localized photogenerated states. This work reveals the central role of localized charge transfer transitions that arise due to cation inversion in ternary iron spinel oxides in the formation of photogenerated polaronic states. Correlating inversion and composition of these ternary materials to their electronic structure is critical for rational material design and offers insights into band-edge engineering to sensitize optically accessible self-trapped excited states.

## Supplementary Material



## Notes

Experimental and computational methods, spin-resolved electronic band structures of iron spinel oxides, X-ray fluorescence spectra, additional powder X-ray diffraction patterns with Rietveld refinements, transmission and reflection spectra used in Fresnel analysis to determine the dielectric spectra, resonance Raman spectral correction methods, additional resonance Raman spectra and thermal difference spectra collected above room temperature (PDF)

## References

[1] Krishnan A., Swarnalal A., Das D., Krishnan M., Saji V. S., Shibli S. M. A. (2024). A Review on Transition Metal Oxides Based Photocatalysts
for Degradation of Synthetic Organic Pollutants. J. Environ. Sci..

[2] Sanchez-Lievanos K. R., Sun T., Gendrich E. A., Knowles K. E. (2024). Surface
Adsorption and Photoinduced
Degradation: A Study of Spinel Ferrite Nanomaterials for Removal of
a Model Organic Pollutant from Water. Chem.
Mater..

[3] Toh R. J., Eng A. Y. S., Sofer Z., Sedmidubsky D., Pumera M. (2015). Ternary Transition Metal Oxide Nanoparticles with Spinel
Structure for the Oxygen Reduction Reaction. ChemElectroChem.

[4] Arif N., Zafar M. N., Batool M., Humayun M., Iqbal M. A., Younis M., Li L., Li K., Zeng Y.-J. (2024). Recent
Advances and Perspectives on Iron-Based Photocatalysts. J. Mater. Chem. C.

[5] Walsh A., Ahn K.-S., Shet S., Huda M. N., Deutsch T. G., Wang H., Turner J. A., Wei S.-H., Yan Y., Al-Jassim M. M. (2009). Ternary
Cobalt Spinel Oxides for Solar Driven Hydrogen
Production: Theory and Experiment. Energy Environ.
Sci..

[6] Chang T.-C., Lu Y.-T., Lee C.-H., Gupta J. K., Hardwick L. J., Hu C.-C., Chen H.-Y. T. (2021). The Effect of Degrees of Inversion
on the Electronic Structure of Spinel NiCo_2_O_4_ : A Density Functional Theory Study. ACS Omega.

[7] Hakim M. A., Haque M. M., Huq M., Nordblad P. (2011). Spin-Glass-Like
Ordering
in the Spinel ZnFe_2_O_4_ Ferrite. Phys. B.

[8] Peddis D., Yaacoub N., Ferretti M., Martinelli A., Piccaluga G., Musinu A., Cannas C., Navarra G., Greneche J. M., Fiorani D. (2011). Cationic Distribution and Spin Canting
in CoFe_2_O_4_ Nanoparticles. J. Phys.: Condens. Matter.

[9] Robertson J. M., Pointon A. J. (1966). The Cation Distribution
in Nickel Ferrite. Solid State Commun..

[10] Restrepo O. A., Arnache Ó., Mousseau N. (2024). An Approach to Understanding the
Formation Mechanism of NiFe_2_O_4_ Inverse Spinel. Materialia.

[11] Mullurkara S., Fang Y., Taddei K. M., Wang G., Ohodnicki P. (2023). Experimental
and Theoretical Investigation of Cation Site Occupation and Magnetic
Ordering in CoFe_2_O_4_. IEEE
Trans. Magn..

[12] Andersen H. L., Saura-Múzquiz M., Granados-Miralles C., Klemmt R., Bøjesen E. D., Christensen M. (2025). Crystal/Magnetic Structure and Cation Inversion in
Hydrothermally Synthesized MnFe_2_O_4_, CoFe_2_O_4_, NiFe_2_O_4_, and ZnFe_2_O_4_ Nanoparticles: A Neutron Powder Diffraction
Study. CrystEngComm.

[13] Andersen H. L., Saura-Múzquiz M., Granados-Miralles C., Canévet E., Lock N., Christensen M. (2018). Crystalline
and Magnetic Structure–Property
Relationship in Spinel Ferrite Nanoparticles. Nanoscale.

[14] Sanchez-Lievanos K. R., Stair J. L., Knowles K. E. (2021). Cation Distribution in Spinel Ferrite
Nanocrystals: Characterization, Impact on Their Physical Properties,
and Opportunities for Synthetic Control. Inorg.
Chem..

[15] Bhargava A., Chen C. Y., Dhaka K., Yao Y., Nelson A., Finkelstein K. D., Pollock C. J., Toroker M. C., Robinson R. D. (2019). Mn Cations
Control Electronic Transport in Spinel Co*
_x_
*Mn_3–*x*
_O_4_ Nanoparticles. Chem. Mater..

[16] Bhargava A., Eppstein R., Sun J., Smeaton M. A., Paik H., Kourkoutis L. F., Schlom D. G., Toroker M. C., Robinson R. D. (2020). Breakdown
of the Small-Polaron Hopping Model in Higher-Order Spinels. Adv. Mater..

[17] Rossman, G. Why Hematite Is Red: Correlation of Optical Absorption Intensities and Magnetic Moments of Fe^3+^ Minerals. InMineral Spectroscopy: A Tribute to Roger G. Burns; Dyar, M. D. , McCamman, C. , Schaefer, M. W. , Eds.; Geochemical Society, 1996; pp 23−27.

[18] Craddock E. P., Shelton J. L., Ruggiero M. T., Knowles K. E. (2025). Local Coordination
Geometry within Cobalt Spinel Oxides Mediates Photoinduced Polaron
Formation. Chem. Sci..

[19] Fritsch D. (2018). Electronic
and Optical Properties of Spinel Zinc Ferrite: *Ab Initio* Hybrid Functional Calculations. J. Phys.:
Condens. Matter.

[20] Li J., Cheepurupalli K. K., English N. J., Bandaru S., Zhang X. (2025). Unraveling
Cation Distribution and Defect Roles in Substituted Ferrite Performance:
An Atomistic DFT Study. J. Appl. Phys..

[21] Munawar H.
B., Hussain A., Gouadria S., Noreen S., Bibi N., Tariq A., Tahir M. B., Rehman J. U., Arshad S., Ali H. E. (2023). Structural,
Electronic, Magnetic, and Optical Properties
of MFe_2_O_4_ (M = Ni, Fe, Co) Spinel Ferrites:
A Density Functional Theory Study. Int. J. Quantum
Chem..

[22] Almutairi T. S. (2025). Unveiling
the Impact of Spin and Cation Dynamics on Raman Spectroscopy in Co-Ferrite. ACS Phys. Chem. Au.

[23] Almutairi T. S. (2023). Unraveling
the Complex Interplay of Phase Transitions in Spinel Ferrites: A Comprehensive
Quantum Mechanical Vibrational Study of ZnFe_2_O_4_. ACS Omega.

[24] Szotek Z., Temmerman W. M., Ködderitzsch D., Svane A., Petit L., Winter H. (2006). Electronic
Structures of Normal and Inverse Spinel
Ferrites from First Principles. Phys. Rev. B.

[25] Shelton J. L., Knowles K. E. (2021). Thermally Activated Optical Absorption into Polaronic
States in Hematite. J. Phys. Chem. Lett..

[26] Shelton J. L., Knowles K. E. (2022). Polaronic Optical Transitions in Hematite (α-Fe_2_O_3_) Revealed by First-Principles Electron–Phonon
Coupling. J. Chem. Phys..

[27] Londo S., Biswas S., Husek J., Pinchuk I. V., Newburger M. J., Boyadzhiev A., Trout A. H., McComb D. W., Kawakami R., Baker L. R. (2020). Ultrafast
Spin Crossover in a Room-Temperature Ferrimagnet:
Element-Specific Spin Dynamics in Photoexcited Cobalt Ferrite. J. Phys. Chem. C.

[28] Londo S., Biswas S., Pinchuk I. V., Boyadzhiev A., Kawakami R. K., Baker L. R. (2022). Ultrafast Optical
Spin Switching
in Ferrimagnetic Nickel Ferrite (NiFe_2_O_4_) Studied
by XUV Reflection–Absorption Spectroscopy. J. Phys. Chem. C.

[29] Restelli S., Cannelli O., Colonna N., Grova C., Usai P., Puppin M., Mensi M., Barantani F., Meng Y., Teyssier J., Oppermann M., Pennacchio F., Bacellar C., Rouxel J. R., Leroy L. D., Dogadov O., Ohannessian N., Pergolesi D., Galinetto P., Piekarz P., Ptok A., Chaudhary S., Fiete G. A., Chergui M., Baldini E., Mancini G. F. (2026). Ultrafast
Formation of Jahn–Teller Polarons Revealed by State-Selective
Excitation in Correlated Spinel Co_3_ O_4_. J. Am. Chem. Soc..

[30] Carneiro L. M., Cushing S. K., Liu C., Su Y., Yang P., Alivisatos A. P., Leone S. R. (2017). Excitation-Wavelength-Dependent Small
Polaron Trapping of Photoexcited Carriers in α-Fe_2_O_3_. Nat. Mater..

[31] Husek J., Cirri A., Biswas S., Baker L. R. (2017). Surface Electron
Dynamics in Hematite (α-Fe_2_O_3_): Correlation
between Ultrafast Surface Electron Trapping and Small Polaron Formation. Chem. Sci..

[32] Li K., Wang Y., Jiang L., Gao G., Wen G., Zhang Y., Wang X., Lou S., Bonn M., Wang H. I., Zhu T. (2025). Unlocking Ultrafast
Hot Hole Transport
in Transition Metal Oxides Governed by the Nature of Optical Transitions. Nat. Commun..

[33] Sachs M., Harnett-Caulfield L., Pastor E., Davies B., Sowood D. J. C., Moss B., Kafizas A., Nelson J., Walsh A., Durrant J. R. (2025). Metal-Centred States Control Carrier
Lifetimes in Transition
Metal Oxide Photocatalysts. Nat. Chem..

[34] Craddock E. P., Knowles K. E. (2026). Signatures of Photogenerated Small Polarons in Transition
Metal Oxides. Chem. Commun..

[35] Sanchez-Lievanos K. R., Knowles K. E. (2022). Controlling Cation
Distribution and Morphology in Colloidal
Zinc Ferrite Nanocrystals. Chem. Mater..

[36] Bhargava A., Chen C. Y., Finkelstein K. D., Ward M. J., Robinson R. D. (2018). X-Ray Emission
Spectroscopy: An Effective Route to Extract Site Occupation of Cations. Phys. Chem. Chem. Phys..

[37] Zeng X., Zhang J., Zhu S., Deng X., Ma H., Zhang J., Zhang Q., Li P., Xue D., Mellors N. J., Zhang X., Peng Y. (2017). Direct Observation
of Cation Distributions of Ideal Inverse Spinel CoFe_2_O_4_ Nanofibres and Correlated Magnetic Properties. Nanoscale.

[38] White W. B., DeAngelis B. A. (1967). Interpretation
of the Vibrational Spectra of Spinels. Spectrochim.
Acta, Part A.

[39] Lazarević Z. Ž., Jovalekić Č., Milutinović A., Sekulić D., Ivanovski V. N., Rečnik A., Cekić B., Romčević N. Ž. (2013). Nanodimensional
Spinel NiFe_2_O_4_ and ZnFe_2_O_4_ Ferrites Prepared by Soft Mechanochemical Synthesis. J. Appl. Phys..

[40] Shannon R. D., Prewitt C. T. (1969). Effective Ionic Radii in Oxides and Fluorides. Acta Crystallogr.,.

[41] Lany S. (2015). Semiconducting
Transition Metal Oxides. J. Phys.: Condens.
Matter.

[42] Rajan R., Craddock E. P., Knowles K. E. (2026). Impact
of Composition on the Optical
Properties of Colloidal Ternary Spinel Oxide Nanocrystals: Spinel
Ferrites versus Spinel Gallates. Chem. Mater..

[43] Saini R. K., Miriyala K., Chernykh D., Engel Y., Rashkovskiy A., Grave D. A. (2026). Intrinsic Charge-Carrier Transport Limitations in ZnFe_2_O_4_ Revealed by Time-Resolved Microwave Conductivity. J. Phys. Chem. Lett..

[44] Granone L. I., Ulpe A. C., Robben L., Klimke S., Jahns M., Renz F., Gesing T. M., Bredow T., Dillert R., Bahnemann D. W. (2018). Effect
of the Degree of Inversion on Optical Properties
of Spinel ZnFe_2_O_4_. Phys.
Chem. Chem. Phys..

