# Supplementary material for: Correlating Composition and Cation Inversion in Ternary Iron Spinel Oxides with Localization of Photoexcited States
Source: J Am Chem Soc. 2026 Jun 25;148(26):27484–93. doi: 10.1021/jacs.6c05073 (PMC13352617; doi:10.1021/jacs.6c05073)
Supplement: Supplementary file 1 [file ja6c05073_si_001.pdf]

Supporting Information

Correlating Composition and Cation Inversion in Ternary Iron Spinel Oxides with Localization of Photoexcited States

Erica P. Craddock[1], William W. Brennessel[1], Michael. T. Ruggiero[1], Kathryn E. Knowles[1,*]

[1]Department of Chemistry, University of Rochester, Rochester, New York 14627, USA

*Corresponding Author e-mail: kknowles@ur.rochester.edu

I. Experimental Methods

Sample Preparation – Ternary iron spinel oxide thin films were prepared using stoichiometric ratios of metal nitrate salts as precursors in a sol-gel process. For $CoFe_2O_4$, 2 mmol of Fe(III) nitrate hexahydrate and 1 mmol of Co(II) nitrate hexahydrate was used; for $NiFe_2O_4$, 2 mmol of Fe(III) nitrate nonahydrate and 1 mmol of Ni(II) nitrate hexahydrate was used and for $FeNi_2O_4$ 2 mmol of Ni(II) nitrate hexahydrate and 1 mmol Fe(III) nitrate nonahydrate was used. These nitrate salts were combined with 4.5 mmol citric acid monohydrate and 4.5 mmol ethylene glycol in 6 mL of ethanol. Aliquots (50 mL) of precursor solutions were spin-coated at 5000 rpm onto sapphire substrates and annealed at 600 °C for 5 minutes. Multiple layers were deposited in this manner before a final annealing at 600 °C for 24 hours followed by cooling to room temperature over 2 hours. One annealed aliquot contributes ~40 nm to film thickness.

Structural Characterization – X-ray diffraction patterns were collected on a Rigaku 3kW (CuKα radiation) SmartLab system with a chi-phi cradle, cross beam optics, and a HyPix-3000 detector. Scans were performed in 2θ in parallel beam mode with grazing incident angles between 0.5-1.5 degrees. Optics included an incident 0.5-degree Soller slit, an incident slit width of 0.5 mm, a length-limiting slit width of 10 mm, and either an initial receiving slit of 1.0 mm and a second receiving slit of 1.1 mm or both receiving slits open and a parallel slit analyzer of 0.5 mm. The detector was operated in 0D mode. Scans were performed from 5 to 70 degrees in 2θ in steps of 0.01 degree at speeds ranging from 4 to 12 degrees per minute. The diffraction patterns were further analyzed with Rietveld refinement using FullProf software. Energy dispersive X-ray emission spectra of each sample were collected in triplicate under vacuum with 3 mm beam collimation using a Shimadzu EDX-8100 instrument.

Optical spectroscopy – Transmission and reflection spectra were collected at with an Agilent Cary 7000 Spectrometer with Universal Measurement Accessory from 2460 – 200 nm. The samples were set to a 10^0 angle and transmission spectra were collected with the detector at 180^0 while reflection spectra were collected with a 20^0 detector angle. Temperature-dependent spectra were collected using a home-built setup in which the sample is sandwiched between two donut-shaped ceramic heaters, which allow optical access through their centers. The temperature was controlled using an Omega temperature controller. Using a previously reported Fresnel analysis method,[1,2] the imaginary dielectric spectra of the samples were calculated at room temperature and elevated temperatures from the measured transmission and reflection spectra.

Raman spectroscopy - Low-frequency Raman spectroscopy experiments (Fig. 3A) were performed using a 785-nm excitation source coupled with a commercial Raman probe module from Coherent. The excitation and scattered radiation were collected using a Nikon LV100 microscope that utilized a 50x polarization-conserving objective, which resulted in a spot size of ~ 4 micrometers. The scattered radiation was dispersed using an Andor Shamrock 500i spectrograph, which consisted of a 1200 l/mm grating blazed at 750 nm, and the radiation was detected using an Andor iDus 416 CCD camera. The resonance Raman spectra profiles (Fig. 7) were collected using CW diode lasers with wavelength emissions of 830 nm (1.49 eV), 785 nm(1.58 eV), 660 nm (1.88 eV), 594 nm (2.09 eV), 561 nm (2.21 eV), 532 nm (2.33 eV), 515 nm (2.41 eV), 491 nm (2.53 eV), 457 nm (2.71 eV) and 405 nm (3.06 eV) and Princeton Instruments monochromators equipped with a CCD detector. Laser power was kept < 32 mW to limit the possibility of sample heating. The Raman shift axis was corrected with cyclohexane spectra. The samples' Raman intensity was corrected for grating efficiency, scattering cross section and the sample's absorption coefficient at each wavelength. See Supplementary Information for more details.

II. Computational Methods

Density Functional Theory - The electronic structures of the spinel iron oxide materials $NiFe_2O_4$ and $CoFe_2O_4$ were computed using density functional theory (DFT) and Hubbard- and Hund-corrected DFT (DFT+*U*+*J*), performed using the pseudopotential plane wave package implemented in Quantum ESPRESSO.[3–5] The U and J parameters were calculated with a linear response method (*vide infra*). High plane wave cut-off energies (≤ 1200 eV) were used for all materials to ensure the total energy and total interatomic forces were converged to 10 meV and 0.10 meV/Å. All ternary iron spinel oxides were sampled over a 5x5x5 k-point grid. Both the Perdew, Burke, and Ernzerh exchange-correlation functional revised for solids (PBEsol)[6,7] and Optimized Norm-Conserving Vanderbilt (ONCV) [8,9] method for pseudopotentials were implemented throughout this work. The alignment of unpaired electron spins on adjacent open-shell *d*-metal ions due to pairwise exchange interactions leads to net magnetic ordering. Ternary iron spinel oxide materials, especially $CoFe_2O_4$ and $NiFe_2O_4$, are known to have Neels ferrimagnetic ordering,[10–13] meaning the unpaired spins of metal ions in O_h sites are aligned while the unpaired spins of metal ions in T_d sites are aligned in the opposite direction to O_h alignment. In addition to imposing ferrimagnetic ordering within the primitive cells, inversion parameters of x = 0.0, 0.5, and 1.0 were imposed on both $CoFe_2O_4$ and $NiFe_2O_4$. For each inversion parameter, iterative geometric relaxations were performed using the Broyden–Fletcher–Goldfarb–Shanno (BFGS) algorithm.[14–16] Electronic bands and projected densities of states were then determined for the relaxed structures by first calculating the self-consistent field (SCF) wavefunctions with associated Hubbard and Hund corrections (*vide infra*) and then performing a Fourier interpolation to a larger grid of k-points. For the dielectric calculations, rigid shifts were applied (+0.3 eV for $CoFe_2O_4$ and +0.2 for $NiFe_2O_4$) to bring the lowest energy transition in the computed dielectric function into alignment with the measured spectrum. In order to fulfill the *f*-sum rule governing total oscillator strength, all computed optical spectra are subsequently renormalized by a factor of $(1-(0.3\text{ eV}/\hbar\omega))$.[17,18] Phonon modes of $NiFe_2O_4$ and $CoFe_2O_4$ with imposed cation inversion of x = 0.5 were calculated with density functional perturbation theory (DFPT) using the PHonon code implemented in Quantum ESPRESSO.[3–5] Starting with the same relaxed ground-state configuration used to calculate the electronic states, the dynamical matrices were evaluated at k-point Γ.

Linear Response Hubbard and Hund Parameters - Converged values for plane wave cutoff and k-point grid were determined using the 14-atom primitive cells of the spinel oxide materials. To calculate the Hubbard *U* (electron correlation correction)[19] and Hund *J* (local magnetization correction)[20] for the open-shell metal ions, a linear response to external perturbation method was used.[19,21] In many Hubbard-corrected computations, a U_{eff} parameter ($U_{eff} = U - J$) is implemented, which combines the electron correlation and local magnetization corrections. The benefit of separately calculating the *U* and *J* parameters using a linear response method is that it provides an intrinsic value that has been shown to generate accurate electronic and vibrational results.[21,22] However, the linear response method for computing Hund's *J* parameter is not applicable to ground states that exhibit a non-zero magnetic moment. In the absence of an external applied magnetic field, the total energy of a system is not variational with its magnetization, and inherently, changing the *J* parameter changes the magnetization of a system during the linear response workflow.[22] To determine the values of the Hubbard and Hund parameters as accurately as possible, an antiferromagnetically aligned ternary iron spinel oxide is used in the linear response method for the materials in this study that exhibit a non-zero net magnetization (*vide infra*), and the net ferrimagnetic alignment is then imposed for the final electronic calculations. This method was used to calculate the Hubbard and Hund parameters for all open-shell ions in the normal and inverted primitive cells. See supplementary information for a full table of calculated Hubbard and Hund parameters.

Table S1. Hubbard and Hund Parameters used for $CoFe_2O_4$ and $NiFe_2O_4$ computations

**$CoFe_2O_4$**		**$NiFe_2O_4$**	
**O_h Fe**		**O_h Fe**	
U: 3.42907223349098	J: 3.20279842615131	U: 3.35520751879551	J: 2.55839168496019
**T_d Fe**		**T_d Fe**	
U: 3.28945579895610	J: 3.27868852458925	U: 3.24468017553923	J: 3.08658116310281
**O_h Co**		**O_h Ni**	
**U: 4.05610186658188**	J: 1.30611748982498	U: 3.90927197010538	J: 1.05338702232615
**T_d Co**		**T_d Ni**	
**U: 3.66540173614086**	J: 1.49302897661083	U: 3.90927197010538	J: 1.05338702232615

With cation inversion, there are many configurations possible that yield the final inversion parameter within a primitive cell. Figure 3 in the main text represents the possible configurations of a ternary spinel oxide unit cell, with a total of 56 atoms. The same logic applies to the 14-atom primitive cell used in the electronic and phonon calculations with DFT+U+J. For example, there are 6 different configurations of the atoms yielding a fully inverted primitive cell of $CoFe_2O_4$ (Table S2). All the configurations listed in Table S2 were calculated using DFT+*U*+*J* and Table S3 lists the total energy of each configuration yielding an inversion parameter of x = 0.5 for both $CoFe_2O_4$ and $NiFe_2O_4$. The total energies calculated for each configuration have near identical energies, demonstrating that the configuration does not influence the total electronic energy of the system.

Table S2. Number of configurations yielding overall cation inversion parameters based on 14-atom primitive cell of ternary iron spinel oxides

$CoFe_2O_4$		$NiFe_2O_4$	
**x**	No. Configurations in primitive cell	**x**	No. Configurations in primitive cell
**0.0**	1	**0.0**	1
**0.5**	8	**0.5**	8
**1.0**	6	**1.0**	6

Table S3. Energy calculations of all configurations of partially inverted $CoFe_2O_4$ and $NiFe_2O_4$ (x = 0.5) primitive cells

Configuration	$CoFe_2O_4$ Total Energy (eV)	$NiFe_2O_4$ Total Energy (eV)
1	-137.804	-142.166
2	-137.804	-142.165
3	-137.804	-142.165
4	-137.804	-142.165
5	-137.804	-142.165
6	-137.804	-142.164
7	-137.804	-142.164
8	-137.804	-142.164
Std. Dev	2.07E-05	6.77E-04

From the configuration calculations (*vide supra*), it is evident the total energy and therefore electronic structure is not directly dependent on the configuration yielding a specific inversion parameter. The electronic structures of $CoFe_2O_4$ and $NiFe_2O_4$ with different degrees of inversion are shown below alongside the primitive cell used for the calculations.

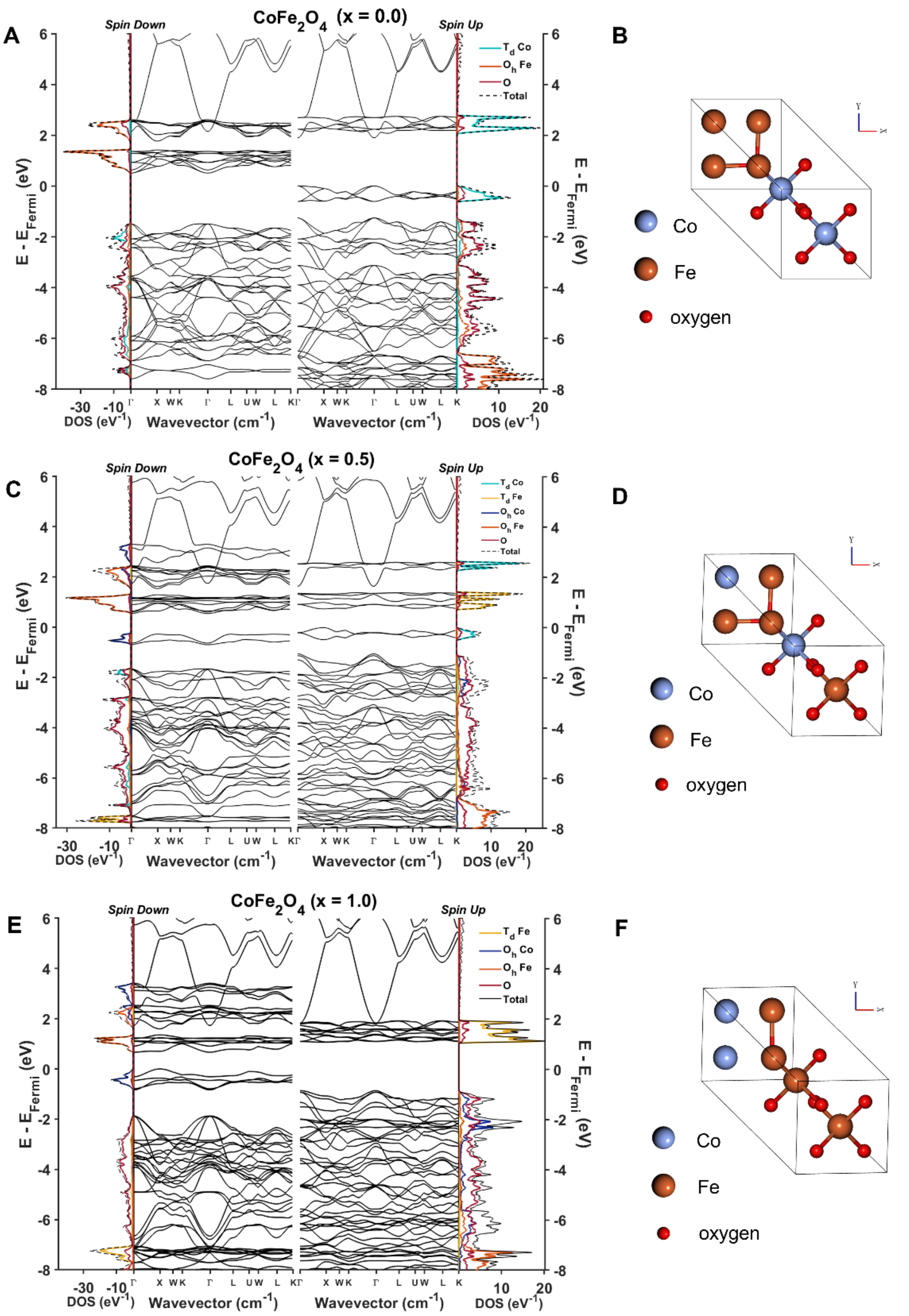

Figure S1. Spin-resolved electronic bands and projected densities of states **(A)** normal $CoFe_2O_4$ (x = 0.0), **(C)** partially inverted $CoFe_2O_4$ (x = 0.5) and **(E)** fully inverted $CoFe_2O_4$ (x = 1.0). The corresponding primitive cell is to the left of the band structure: **(B)** normal $CoFe_2O_4$, **(D)** partially inverted and **(F)** fully inverted.

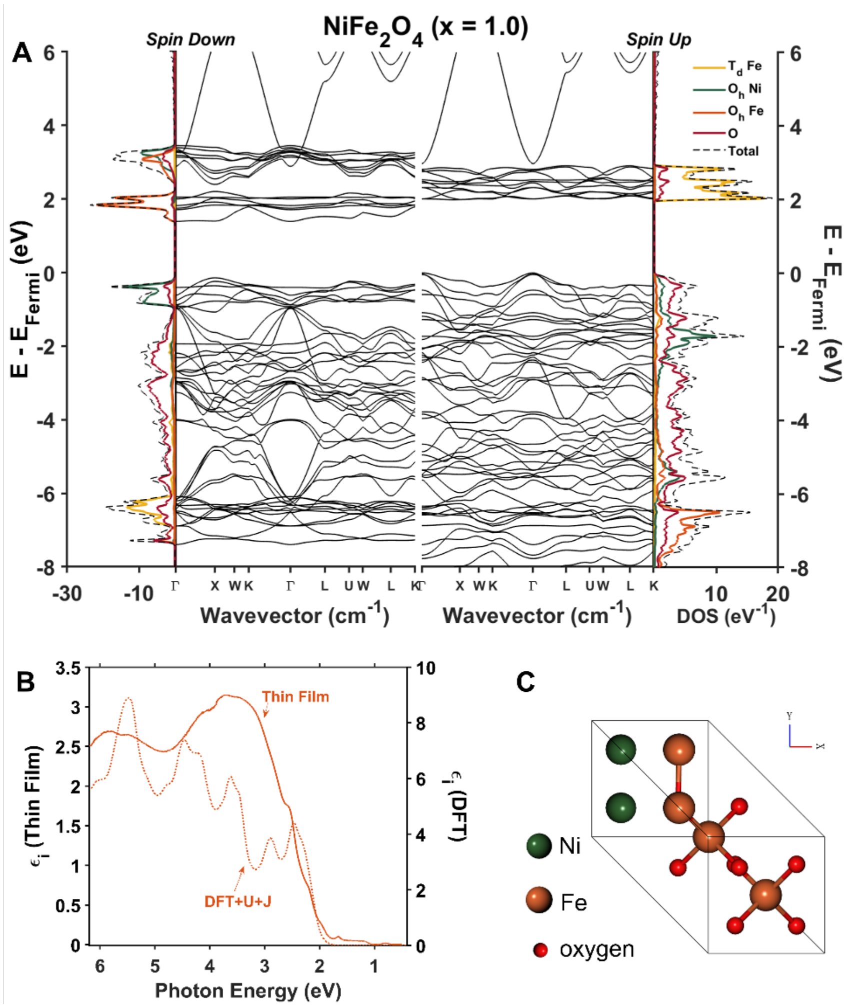

Figure S2. A) Spin-resolved electronic bands and projected densities of states of inverted $NiFe_2O_4$ (x = 1.0), **(B)** the calculated imaginary dielectric spectrum (dashed line) of inverted $NiFe_2O_4$ (x = 1.0) overlaid with the experimental spectrum (solid line) and **(C)** the associated primitive cell of the computations in **(A)** and **(B)**.

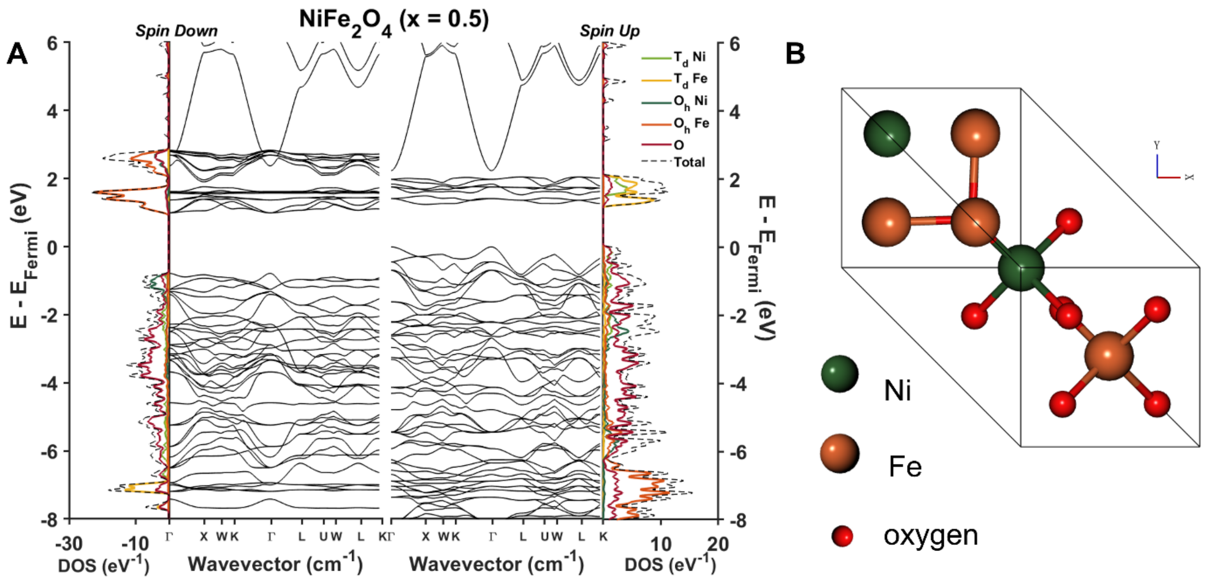

Figure S3. A) Spin-resolved electronic bands and projected densities of states of partially inverted $NiFe_2O_4$ (x = 0.5) and **(B)** the associated primitive cell.

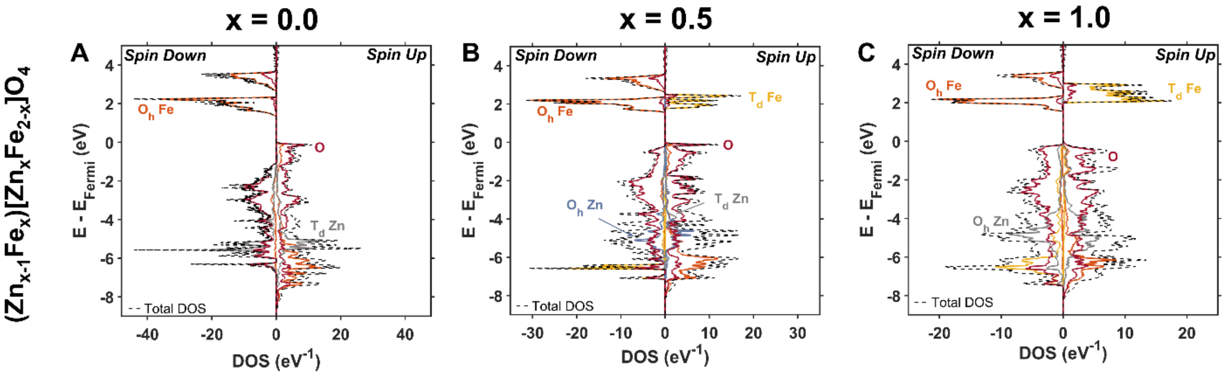

Figure S4. Spin-resolved electronic densities of states of $ZnFe_2O_4$ with inversion parameters of **(A)** x = 0.0, **(B)** x = 0.5 and **(C)** x = 1.0.

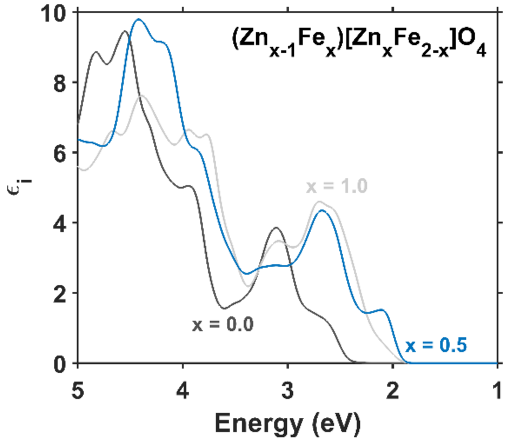

Figure S5. Computed imaginary dielectric spectra of $ZnFe_2O_4$ with varied inversion parameters: x = 0.0 (dark gray), x = 0.5 (blue) and x = 1.0 (light gray).

II. Sample Characterization

To determine the metal:metal ratios of the iron spinel oxide thin films, X-ray fluorescence spectra were collected. Figure S6 plots the spectra which were used to establish the ratios reported in Table 1 of the main text. The pXRD patterns of the iron spinel oxides films were refined with Rietveld analysis using FullProf and are shown in Figure S7. These fittings were used to determine the lattice parameters reported in the main text, Table 1.

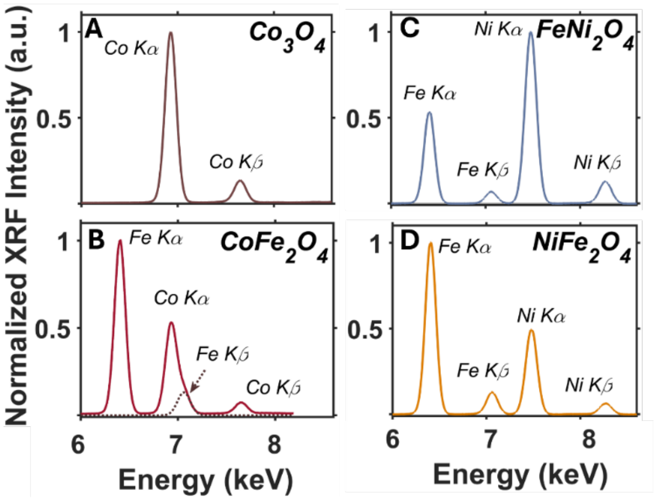

Figure S6. X-ray fluorescence spectra of **(A)** Co_3O_4, **(B)** $CoFe_2O_4$, **(C)** $FeNi_2O_4$ and **(D)** $NiFe_2O_4$ films annealed on sapphire. The Co_3O_4 data are reproduced with permission from ref. 23. Available under a CC BY 3.0 license. Copyright 2025 Royal Society of Chemistry.

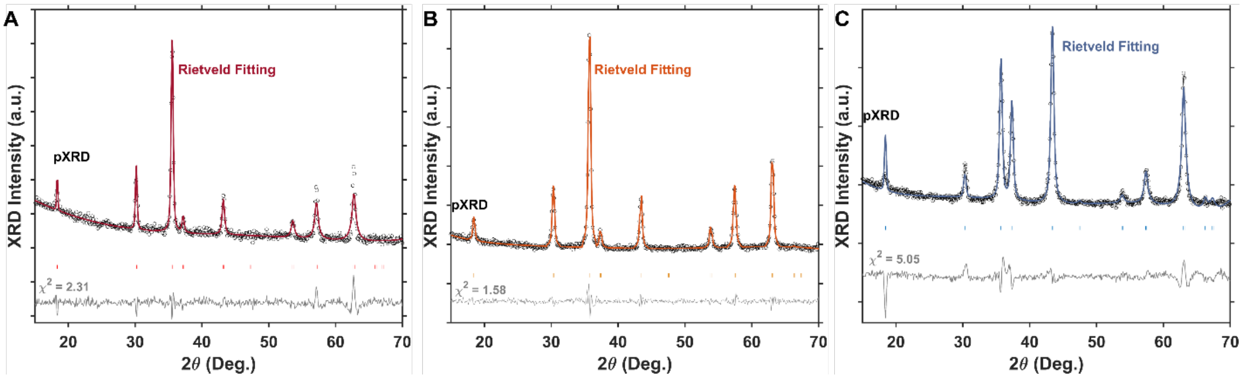

Figure S7. Rietveld-refined pXRD patterns of **(A)** $CoFe_2O_4$, **(B)** $NiFe_2O_4$ and **(C)** $FeNi_2O_4$ films anneal on sapphire. These films were used for Raman collection plotted in the main text (Figure 2, Figure 7, Figure S10).

The imaginary dielectric spectra of $CoFe_2O_4$, $NiFe_2O_4$ and $FeNi_2O_4$ were determined experimentally using Fresnel analysis methods, previously reported.[1,2] The Raman analysis required thick films (d > 400 nm) to ensure optimal scattering off the sample; however, this led to high opacity across the optical spectrum (Fig. S8). To calculate the imaginary dielectric, thin films with thicknesses less than 150 nm and low opacity across the optical spectrum were used. Figures S9 – S11 show the transmission and reflection of these films used in the Fresnel analysis to calculate the associated imaginary dielectric spectra of $CoFe_2O_4$, $NiFe_2O_4$ and $FeNi_2O_4$ (Main Text Fig. 4-5). Alongside the optical spectra are the associated pXRD patterns and XRF spectra for $CoFe_2O_4$, $NiFe_2O_4$ and $FeNi_2O_4$ samples.

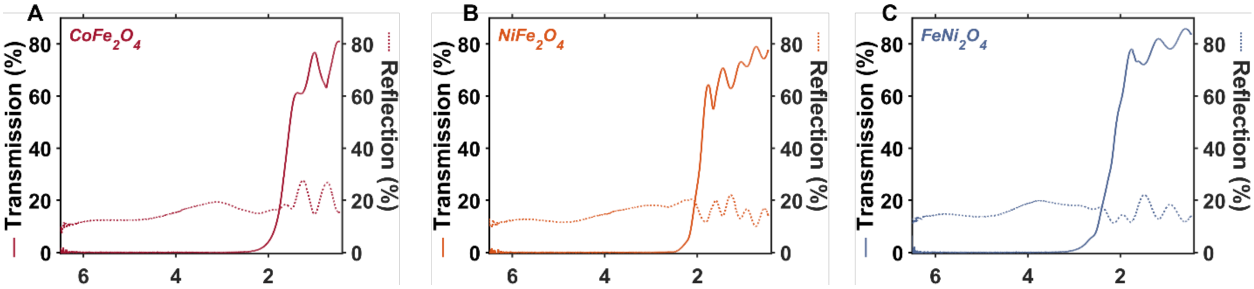

Figure S8. Transmission and reflection spectra of iron spinel oxides used for Raman and powder XRD data shown in Figure 3 of main text.

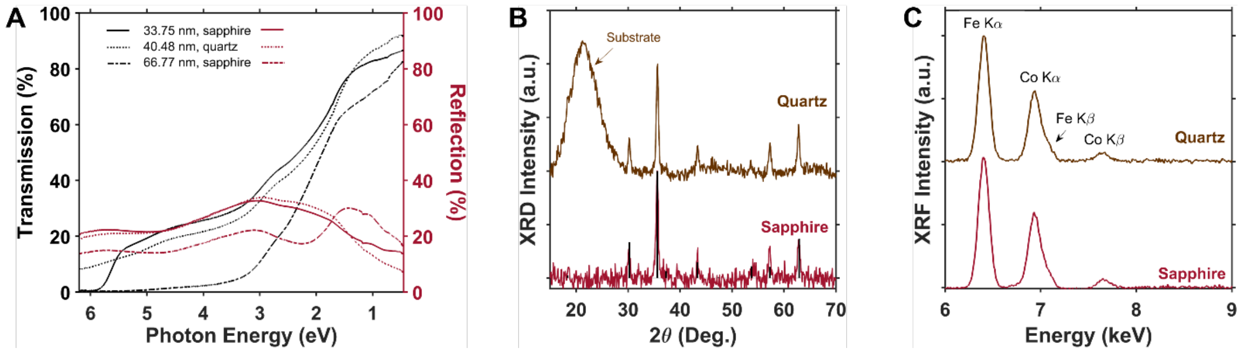

Figure S9. A) Transmission and reflection spectra of $CoFe_2O_4$ thin film samples used for determining experimental dielectric spectrum with Fresnel analysis. **B)** XRD pattern of thin films of sapphire and quartz substrates overlaid with ICDD 03-0864 and **(C)** XRF spectra of the thin films in **(A)**. It should be noted that only the 66 nm-thick film on sapphire is shown in **(B)** and **(C)**.

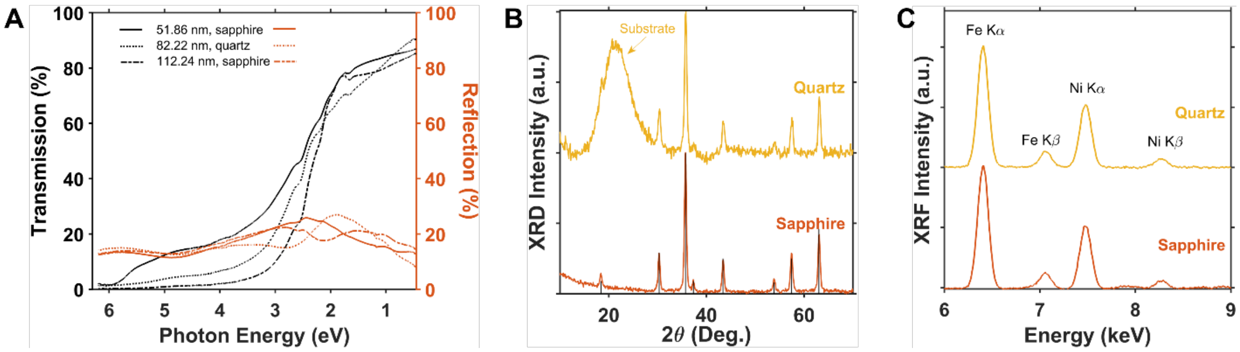

Figure S10. A) Transmission and reflection spectra of $NiFe_2O_4$ thin film samples used for determining experimental dielectric spectrum with Fresnel analysis. **B)** XRD pattern of thin films of sapphire and quartz substrates overlaid with ICDD 54-0964 and **(C)** XRF spectra of the thin films in **(A)**. It should be noted that only the 112 nm-thick film on sapphire is shown in **(B)** and **(C)**.

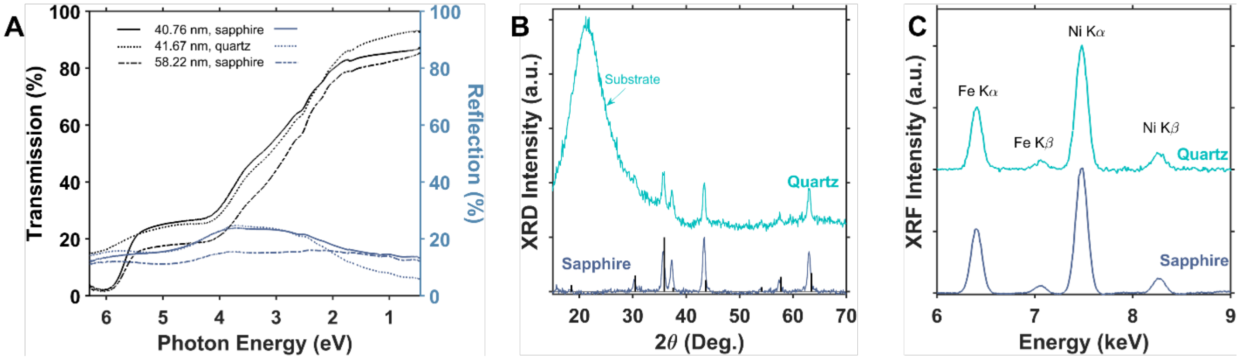

Figure S11. A) Transmission and reflection spectra of $FeNi_2O_4$ thin film samples used for determining experimental dielectric spectrum with Fresnel analysis. **B)** XRD pattern of thin films of sapphire and quartz substrates overlaid with ICDD 01-074-6507 and **(C)** XRF spectra of the thin films in **(A)**. It should be noted that only the 58 nm-thick film on sapphire is shown in **(B)** and **(C)**.

III. Raman spectra corrections

The Raman spectra were collected and corrected based on the same protocol in our previous work.[23] The CCD x-axis was corrected for Raman shift using cyclohexane as a standard and correcting the associated pixels with a second order polynomial regression fitting. Raman spectra were corrected for grating and CCD efficiency, scattering efficiency, and sample absorption. Published grating and CCD efficiencies for the Princeton Monochromators were used for instrumental corrections. To correct for scattering efficiency of the samples, cyclohexane was collected after each sample scan. The prominent peak at 100 meV was integrated for each laser wavelength for the same accumulation time and compared to the theoretical value of the intensity of scattered light expected for each laser wavelength (Eq S1). From this ratio, we obtained a correction factor for each excitation wavelength that accounts for variations in laser power, solid angle collection, etc. Applying these correction factors to the regular Raman spectrum of cyclohexane recovers the expected λ^{-4} dependence of the scattering efficiency (Figure S12A), confirming the efficacy of this correction procedure. The resonance Raman spectra are corrected with the scattering efficiency determined from cyclohexane and then further corrected for the differences in sample absorption at the various excitation wavelengths by dividing by the absorption coefficient at the corresponding photon excitation wavelength. Figure S12 plots the absorption coefficient of (B) $CoFe_2O_4$, (C) $NiFe_2O_4$ and (D) $FeNi_2O_4$.

$$\text{Scat. Efficiency} = \text{Integrated area} / (h\nu)^4 \qquad \text{(S1)}$$

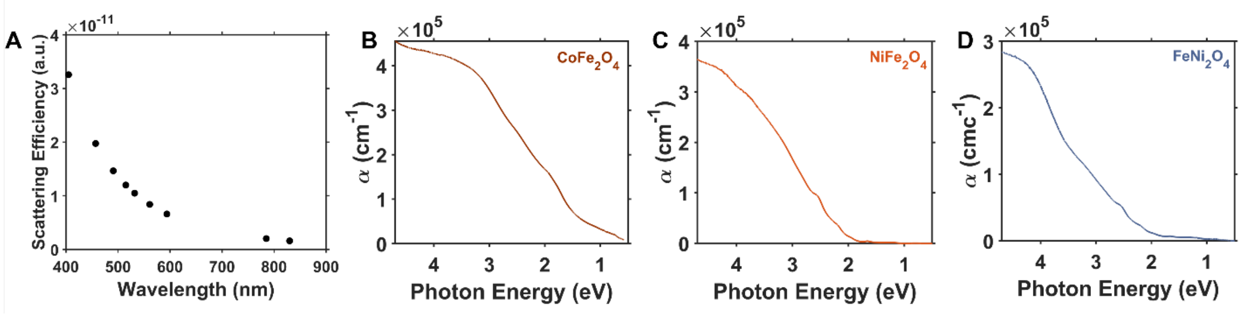

Figure S12. (A) scattering efficiency curve determined from cyclohexane spectra collected at each associated wavelength. Absorption coefficient spectra of **(B)** $CoFe_2O_4$, **(C)** $NiFe_2O_4$ and **(D)** $FeNi_2O_4$.

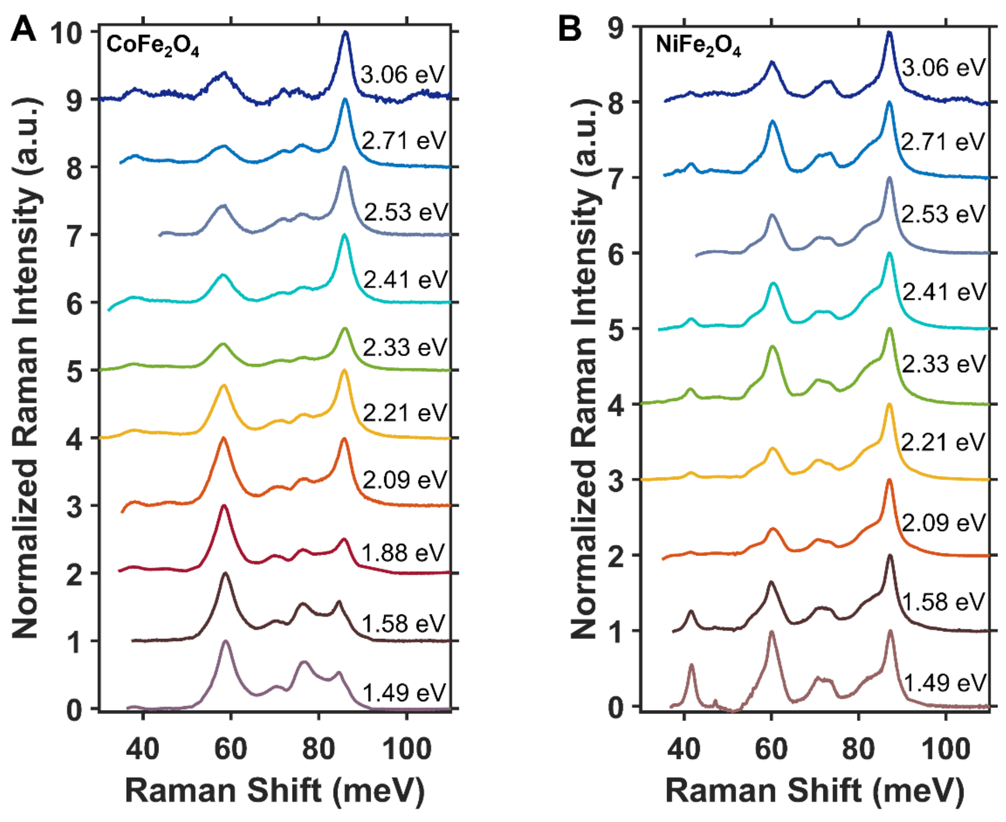

Figure S13. Full resonance Stokes Raman profiles of **(A)** $CoFe_2O_4$ and **(B)** $NiFe_2O_4$ polycrystalline thin films annealed on sapphire with excitation photon energies from 3.06 to 1.49 eV.

The analogous Stokes Raman spectra collection was performed on $FeNi_2O_4$ as reported in the main text on $CoFe_2O_4$ and $NiFe_2O_4$ (Fig. 6). Figure S14 plots the Stokes Raman spectra of $FeNi_2O_4$ collected with photon excitation energy sources ranging from 3.06 – 1.58 eV. It should be noted that collection of $FeNi_2O_4$ with 1.49 eV excitation was attempted, but substrate signal dominated the spectrum, masking sample signal, especially the higher energy phonon modes. The strong enhancement of the 86-meV phonon mode at 1.58-eV excitation in $FeNi_2O_4$ is evidence of a strongly phonon-coupled optical transition. The trend of strong phonon coupling at 1.58 eV is further corroborated with a replicated excitation profile of $FeNi_2O_4$ annealed on quartz, ruling out the influence of substrate effects (Fig. S15). By comparison to $NiFe_2O_4$, the Raman spectrum of $FeNi_2O_4$ is nearly indistinguishable and tracing the enhancement phonon modes with respect to excitation energy, it is clear the 86-meV phonon mode in both $NiFe_2O_4$ and $FeNi_2O_4$ is most enhanced at 1.58 eV. The energy of the MMCT transitions observed in the dielectric spectra of $NiFe_2O_4$ and $FeNi_2O_4$ are the same (Fig. 5, Fig. S13), and comparing the thermal difference dielectric spectrum of both Fe-Ni spinel oxides, it is evident the MMCT transitions in both materials exhibit temperature dependence (Fig. S14). With combined evidence from resonance Raman and thermal difference spectroscopies, it is evident the Ni:Fe ratio does not directly influence phonon-coupled optical transitions in ternary spinel oxides. Rather, the MMCT transitions that arise as function of cation distribution of nickel and iron within the spinel structure of the two materials influences the phonon mode enhancement.

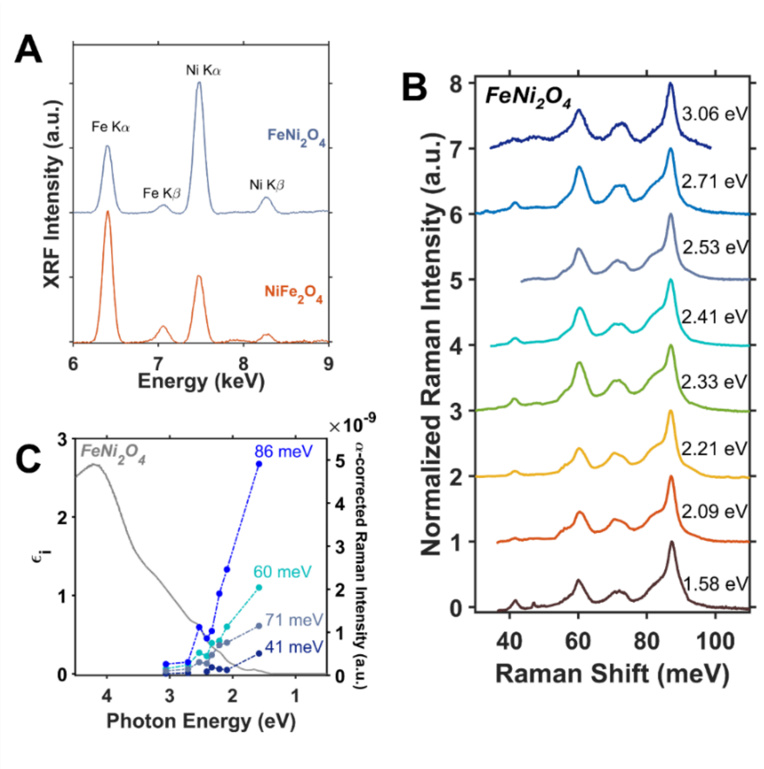

Figure S14. A) X-ray fluorescence spectra of $FeNi_2O_4$ (blue) compared to $NiFe_2O_4$ (orange). **B)** Stokes resonance Raman of $FeNi_2O_4$ polycrystalline thin film with excitation photon energies from 3.06 to 1.58 eV. **C)** Imaginary dielectric spectrum of $FeNi_2O_4$ overlaid with Raman mode intensities corrected for scattering cross section and sample absorption.

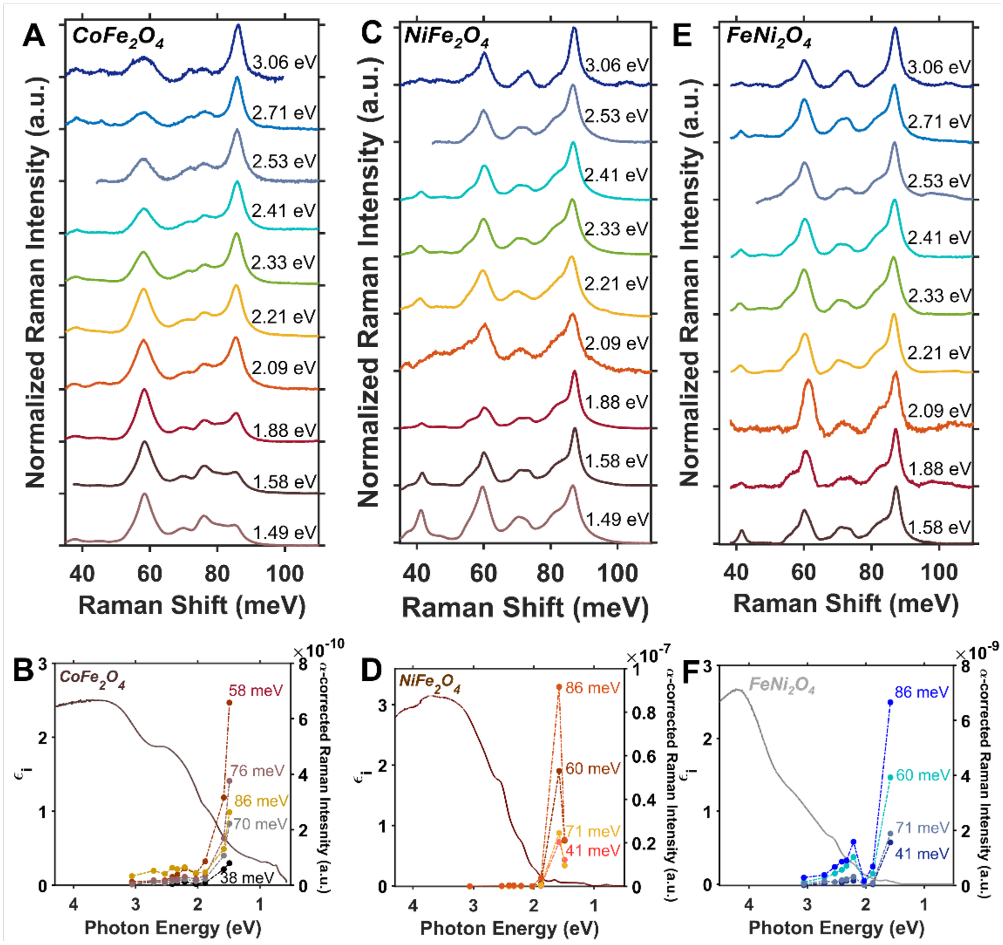

Figure S15. Resonance Stokes Raman spectra profiles and associated phonon intensities overlaid with imaginary dielectric spectrum of the iron spinel thin films **(A-B)** $CoFe_2O_4$, **(C-D)** $NiFe_2O_4$ and **(E-F)** $FeNi_2O_4$ annealed on quartz substrates.

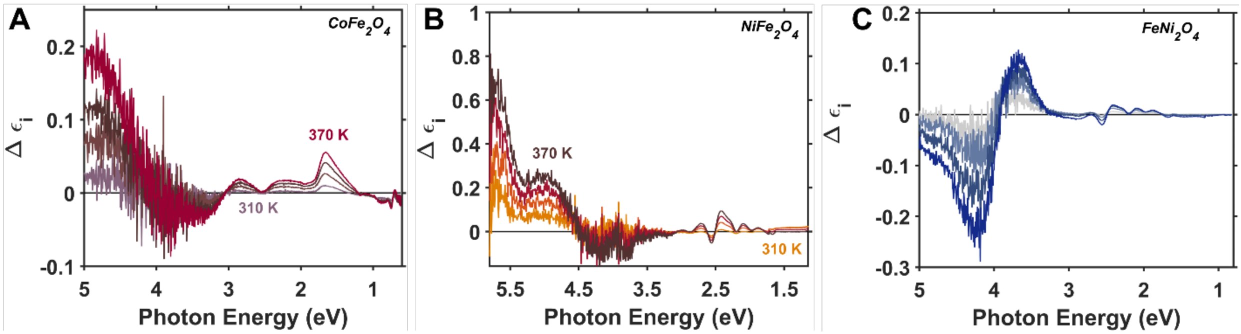

Figure S16. Thermal difference imaginary dielectric spectra of **(A)** $CoFe_2O_4$, **(B)** $NiFe_2O_4$ and **(C)** $FeNi_2O_4$ above room temperature (310 – 370 K) relative to room temperature (293 K).

References

(1) Shelton, J. L.; Knowles, K. E. Thermally Activated Optical Absorption into Polaronic States in Hematite. *J. Phys. Chem. Lett.* **2021**, *12* (13), 3343–3351. https://doi.org/10.1021/acs.jpclett.0c03751.

(2) Barybin, A.; Shapovalov, V. Substrate Effect on the Optical Reflectance and Transmittance of Thin-Film Structures. *International Journal of Optics* **2010**, *2010*, 1–18. https://doi.org/10.1155/2010/137572.

(3) Giannozzi, P.; Baroni, S.; Bonini, N.; Calandra, M.; Car, R.; Cavazzoni, C.; Ceresoli, D.; Chiarotti, G. L.; Cococcioni, M.; Dabo, I.; Dal Corso, A.; De Gironcoli, S.; Fabris, S.; Fratesi, G.; Gebauer, R.; Gerstmann, U.; Gougoussis, C.; Kokalj, A.; Lazzeri, M.; Martin-Samos, L.; Marzari, N.; Mauri, F.; Mazzarello, R.; Paolini, S.; Pasquarello, A.; Paulatto, L.; Sbraccia, C.; Scandolo, S.; Sclauzero, G.; Seitsonen, A. P.; Smogunov, A.; Umari, P.; Wentzcovitch, R. M. QUANTUM ESPRESSO: A Modular and Open-Source Software Project for Quantum Simulations of Materials. *J. Phys.: Condens. Matter* **2009**, *21* (39), 395502. https://doi.org/10.1088/0953-8984/21/39/395502.

(4) Giannozzi, P.; Andreussi, O.; Brumme, T.; Bunau, O.; Buongiorno Nardelli, M.; Calandra, M.; Car, R.; Cavazzoni, C.; Ceresoli, D.; Cococcioni, M.; Colonna, N.; Carnimeo, I.; Dal Corso, A.; De Gironcoli, S.; Delugas, P.; DiStasio, R. A.; Ferretti, A.; Floris, A.; Fratesi, G.; Fugallo, G.; Gebauer, R.; Gerstmann, U.; Giustino, F.; Gorni, T.; Jia, J.; Kawamura, M.; Ko, H.-Y.; Kokalj, A.; Küçükbenli, E.; Lazzeri, M.; Marsili, M.; Marzari, N.; Mauri, F.; Nguyen, N. L.; Nguyen, H.-V.; Otero-de-la-Roza, A.; Paulatto, L.; Poncé, S.; Rocca, D.; Sabatini, R.; Santra, B.; Schlipf, M.; Seitsonen, A. P.; Smogunov, A.; Timrov, I.; Thonhauser, T.; Umari, P.; Vast, N.; Wu, X.; Baroni, S. Advanced Capabilities for Materials Modelling with Quantum ESPRESSO. *J. Phys.: Condens. Matter* **2017**, *29* (46), 465901. https://doi.org/10.1088/1361-648X/aa8f79.

(5) Giannozzi, P.; Baseggio, O.; Bonfà, P.; Brunato, D.; Car, R.; Carnimeo, I.; Cavazzoni, C.; De Gironcoli, S.; Delugas, P.; Ferrari Ruffino, F.; Ferretti, A.; Marzari, N.; Timrov, I.; Urru, A.; Baroni, S. Q UANTUM ESPRESSO toward the Exascale. *The Journal of Chemical Physics* **2020**, *152* (15), 154105. https://doi.org/10.1063/5.0005082.

(6) Perdew, J. P.; Burke, K.; Ernzerhof, M. Generalized Gradient Approximation Made Simple. *Phys. Rev. Lett.* **1996**, *77* (18), 3865–3868. https://doi.org/10.1103/PhysRevLett.77.3865.

(7) Perdew, J. P.; Ruzsinszky, A.; Csonka, G. I.; Vydrov, O. A.; Scuseria, G. E.; Constantin, L. A.; Zhou, X.; Burke, K. Restoring the Density-Gradient Expansion for Exchange in Solids and Surfaces. *Phys. Rev. Lett.* **2008**, *100* (13), 136406. https://doi.org/10.1103/PhysRevLett.100.136406.

(8) Hamann, D. R. Optimized Norm-Conserving Vanderbilt Pseudopotentials. *Phys. Rev. B* **2013**, *88* (8), 085117. https://doi.org/10.1103/PhysRevB.88.085117.

(9) Van Setten, M. J.; Giantomassi, M.; Bousquet, E.; Verstraete, M. J.; Hamann, D. R.; Gonze, X.; Rignanese, G.-M. The PseudoDojo: Training and Grading a 85 Element Optimized Norm-Conserving Pseudopotential Table. *Computer Physics Communications* **2018**, *226*, 39–54. https://doi.org/10.1016/j.cpc.2018.01.012.

(10) Wangchhuk, J.; Meher, S. R. Structural, Electronic and Magnetic Properties of Inverse Spinel $NiFe_2O_4$: DFT + U Investigation. *Physics Letters A* **2022**, *443*, 128202. https://doi.org/10.1016/j.physleta.2022.128202.

(11) Mullurkara, S.; Fang, Y.; Taddei, K. M.; Wang, G.; Ohodnicki, P. Experimental and Theoretical Investigation of Cation Site Occupation and Magnetic Ordering in $CoFe_2$ O_4. *IEEE Trans. Magn.* **2023**, *59* (11), 1–5. https://doi.org/10.1109/TMAG.2023.3294018.

(12) Ugendar, K.; Samanta, S.; Rayaprol, S.; Siruguri, V.; Markandeyulu, G.; Nanda, B. R. K. Effect of Frustrated Exchange Interactions and Spin-Half-Impurity on the Electronic Structure of Strongly Correlated NiFe 2 O 4. *Phys. Rev. B* **2017**, *96* (3), 035138. https://doi.org/10.1103/PhysRevB.96.035138.
(13) Peddis, D.; Yaacoub, N.; Ferretti, M.; Martinelli, A.; Piccaluga, G.; Musinu, A.; Cannas, C.; Navarra, G.; Greneche, J. M.; Fiorani, D. Cationic Distribution and Spin Canting in $CoFe_2O_4$ Nanoparticles. *J. Phys.: Condens. Matter* **2011**, *23* (42), 426004. https://doi.org/10.1088/0953-8984/23/42/426004.
(14) *Practical Methods of Optimization*, 2nd ed.; Fletcher, R., Ed.; Wiley: Chichester New York, 2010.
(15) Billeter, S. R.; Turner, A. J.; Thiel, W. Linear Scaling Geometry Optimisation and Transition State Search in Hybrid Delocalised Internal Coordinates. *Phys. Chem. Chem. Phys.* **2000**, *2* (10), 2177–2186. https://doi.org/10.1039/a909486e.
(16) Billeter, S. R.; Curioni, A.; Andreoni, W. Efficient Linear Scaling Geometry Optimization and Transition-State Search for Direct Wavefunction Optimization Schemes in Density Functional Theory Using a Plane-Wave Basis. *Computational Materials Science* **2003**, *27* (4), 437–445. https://doi.org/10.1016/S0927-0256(03)00043-0.
(17) Zacharias, M.; Patrick, C. E.; Giustino, F. Stochastic Approach to Phonon-Assisted Optical Absorption. *Phys. Rev. Lett.* **2015**, *115* (17), 177401. https://doi.org/10.1103/PhysRevLett.115.177401.
(18) Kang, Y.; Peelaers, H.; Krishnaswamy, K.; Van De Walle, C. G. First-Principles Study of Direct and Indirect Optical Absorption in BaSnO3. *Applied Physics Letters* **2018**, *112* (6), 062106. https://doi.org/10.1063/1.5013641.
(19) Cococcioni, M.; De Gironcoli, S. Linear Response Approach to the Calculation of the Effective Interaction Parameters in the LDA + U Method. *Phys. Rev. B* **2005**, *71* (3), 035105. https://doi.org/10.1103/PhysRevB.71.035105.
(20) Himmetoglu, B.; Wentzcovitch, R. M.; Cococcioni, M. First-Principles Study of Electronic and Structural Properties of CuO. *Phys. Rev. B* **2011**, *84* (11), 115108. https://doi.org/10.1103/PhysRevB.84.115108.
(21) Shelton, J. L.; Knowles, K. E. Polaronic Optical Transitions in Hematite (α-Fe_2O_3) Revealed by First-Principles Electron–Phonon Coupling. *The Journal of Chemical Physics* **2022**, *157* (17), 174703. https://doi.org/10.1063/5.0116233.
(22) Himmetoglu, B.; Floris, A.; De Gironcoli, S.; Cococcioni, M. Hubbard-Corrected DFT Energy Functionals: The LDA+U Description of Correlated Systems. *Int. J. Quantum Chem.* **2014**, *114* (1), 14–49. https://doi.org/10.1002/qua.24521.
(23) Craddock, E. P.; Shelton, J. L.; Ruggiero, M. T.; Knowles, K. E. Local Coordination Geometry within Cobalt Spinel Oxides Mediates Photoinduced Polaron Formation. *Chem. Sci.* **2025**, *16* (24), 10759–10770. https://doi.org/10.1039/D5SC01909E.
